# Advancing CRISPR genome editing into gene therapy clinical trials: progress and future prospects

**DOI:** 10.1017/erm.2025.10

**Published:** 2025-03-31

**Authors:** Busra Cetin, Fulya Erendor, Yunus Emre Eksi, Ahter D. Sanlioglu, Salih Sanlioglu

**Affiliations:** Department of Gene and Cell Therapy, Faculty of Medicine, Akdeniz University, Antalya, Turkey

**Keywords:** CRISPR/Cas, genetic diseases, gene editing, gene therapy, medical genetics

## Abstract

Genome editing has recently evolved from a theoretical concept to a powerful and versatile set of tools. The discovery and implementation of CRISPR-Cas9 technology have propelled the field further into a new era. This RNA-guided system allows for specific modification of target genes, offering high accuracy and efficiency. Encouraging results are being announced in clinical trials employed in conditions like sickle cell disease (SCD) and transfusion-dependent beta-thalassaemia (TDT). The path finally led the way to the recent FDA approval of the first gene therapy drug utilising the CRISPR/Cas9 system to edit autologous CD34+ haematopoietic stem cells in SCD patients (Casgevy). Ongoing research explores the potential of CRISPR technology for cancer therapies, HIV treatment and other complex diseases. Despite its remarkable potential, CRISPR technology faces challenges such as off-target effects, suboptimal delivery systems, long-term safety concerns, scalability, ethical dilemmas and potential repercussions of genetic alterations, particularly in the case of germline editing. Here, we examine the transformative role of CRISPR technologies, including base editing and prime editing approaches, in modifying the genetic and epigenetic codes in the human genome and provide a comprehensive focus, particularly on relevant clinical applications, to unlock the full potential and challenges of gene editing.

## Introduction

Genome editing, or gene editing, holds significant promise for preventing and treating human diseases, constituting a remarkable example of how basic research together with applied biotechnology can provide great utility in effectively addressing human pathologies at the very centre (Ref 1). Scientists now understand how single-gene products, even minor nucleotide changes in specific genes, as well as complex interactions between multiple genes and environmental factors, can contribute to the development of various devastating diseases. With this growing knowledge, advanced genome-editing tools have emerged, allowing for precise modifications to the human genome. Powerful tools for targeted genome editing are at hand today to address these pathologies by introducing specific alterations to the human genome through addition, excision, or modification of human genes.

### Targeted genome editing platforms

**Figure 1.**
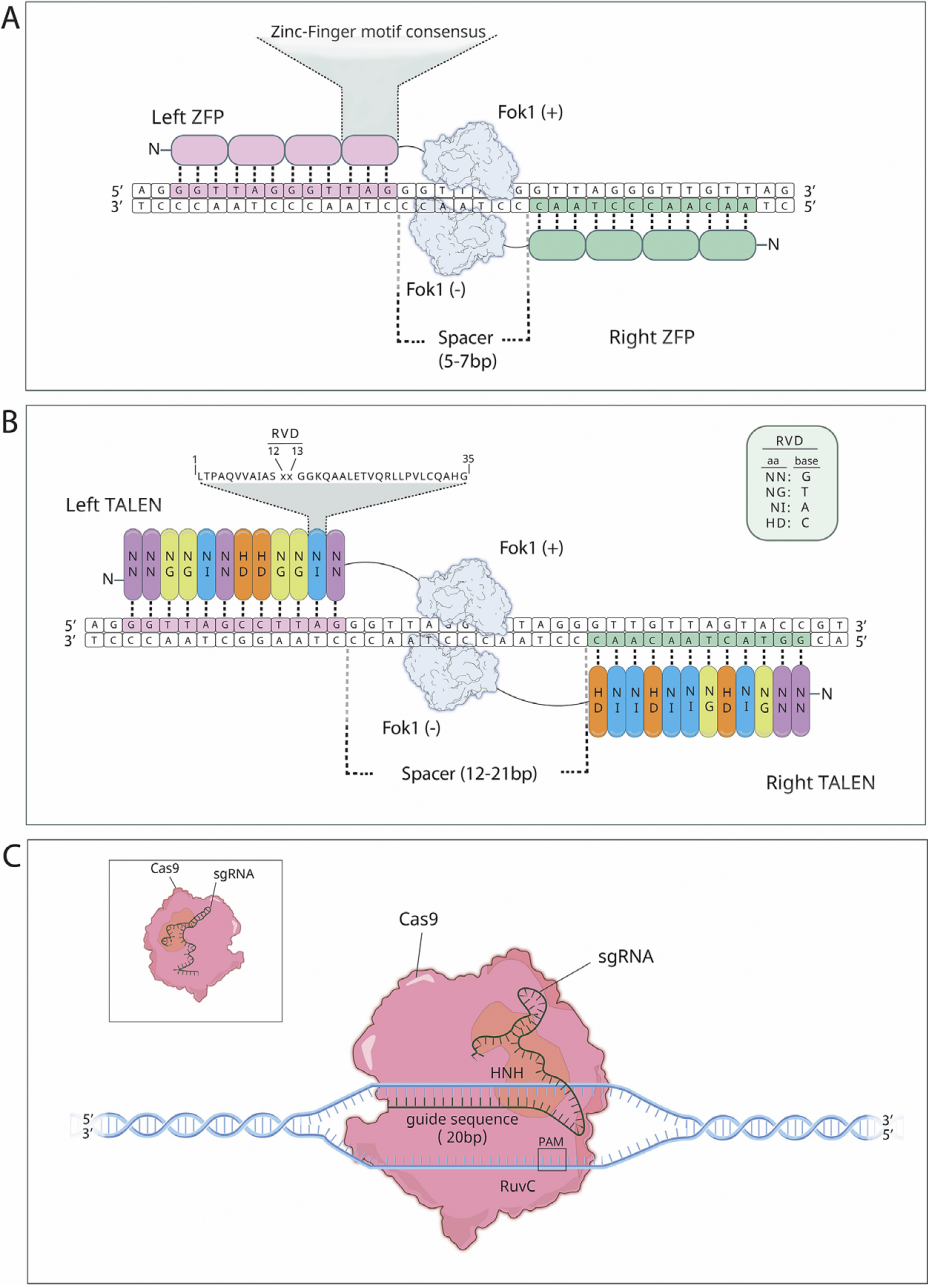
The structure and mechanism of action of the most commonly used programmable nucleases (Ref 3). (a) Zinc-Finger Nucleases (ZFNs). (b) Transcription Activator–Like Effector Nucleases (TALENs). (c) Clustered Regularly Interspaced Short Palindromic Repeats (CRISPR) and CRISPR-Associated protein 9 (CRISPR-Cas9).

Targeted genome editing is a dynamic field of groundbreaking research with great clinical promise. Recent years have witnessed the development of several of these technologies utilising programmable nucleases, with zinc-finger nucleases (ZFNs), transcription activator-like effector nucleases (TALENs), and the RNA-guided CRISPR-Cas nuclease systems constituting the three foundational platforms (Figure 1) (Refs 2–4). Programmable nucleases enhance homologous recombination efficiency by at least 100-fold and/or activate the error-prone non-homologous end joining (NHEJ) mechanism (Ref 5). ZFNs and TALENs employ a strategy involving the attachment of endonuclease catalytic domains to modular DNA-binding proteins to generate targeted double-strand breaks (DSBs) at specific sites in the genome. On the other hand, CRISPR-Cas systems use nucleases guided by small RNAs that engage in Watson-Crick base pairing with the target DNA to introduce DSBs at specific sites for correction (Figure 1c) (Ref 6). CRISPR-Cas-based approaches have recently evolved into base-editing and prime-editing technologies, also presenting a remarkable potential as valuable therapeutic tools that do not involve DSB formation.

#### ZFNs and TALENs

*ZFNs.* The first widespread use of programmable nucleases involved ZFNs, derived from *Xenopus laevis*, the African clawed frog (Ref 7). ZFNs have a modular structure with two main components: a DNA-binding zinc-finger protein (ZFP) domain and a FokI restriction enzyme-derived nuclease domain (Figure 1a). The process of DNA cleavage by ZFNs relies on dimerisation of the FokI nuclease domain, by a collaboration of two ZFN monomers creating an active nuclease. This dimerisation requirement effectively extends the length of recognition sites, greatly improving the precision of ZFNs, although unintended off-target effects also occur. The sequence specificity of ZFNs is controlled by zinc finger proteins (ZFPs), which consist of consecutive arrays of C_2_H_2_ zinc fingers, the commonly found DNA-binding motifs in eukaryotes. Each zinc finger recognises a 3-base pair DNA sequence, and typically 3 to 6 zinc fingers construct an individual ZFN subunit capable of binding to 9 to 18 base pair-long DNA sequences (Ref 8). Constructing zinc finger domains to bind extensive nucleotide stretches with high affinity lacks a straightforward approach. Additionally, commercial ZFN modules are costly, and challenges arise in replacing large fragments, which is crucial for inducible knockouts.

*TALENs.* TALENs emerged as an alternative to the ZFN system (Ref 9). They share a general structural organisation with ZFNs, featuring the FokI nuclease domain at their carboxyl termini. However, TALENs employ a distinct class of DNA-binding domains known as transcription activator-like effectors (TALEs), which are derived from plant pathogenic bacteria *Xanthomonas* spp. (Figure 1b). TALEs consist of consecutive arrays of 33–35 amino acid repeats; each repeat recognises a single base pair within the major groove. The nucleotide specificity within each repeat domain is determined by the repeat variable diresidues (RVDs) located at positions 12 and 13, with four commonly used RVD modules-Asn-Asn, Asn-Ile, His-Asp, and Asn-Gly-corresponding to the recognition of guanine, adenine, cytosine and thymine, respectively. Constructing DNA segments encoding TALE arrays presents challenges due to the potential complexity. TALENs often consist of up to 20 RVDs, and the risk of recombination between the highly homologous sequences makes the process both demanding and time-consuming. Studies continue to reduce the time required to develop genetic constructs expressing TALENs and the complexity of the technique (Ref 10).

#### CRISPR/Cas gene editing systems

**Figure 2.**
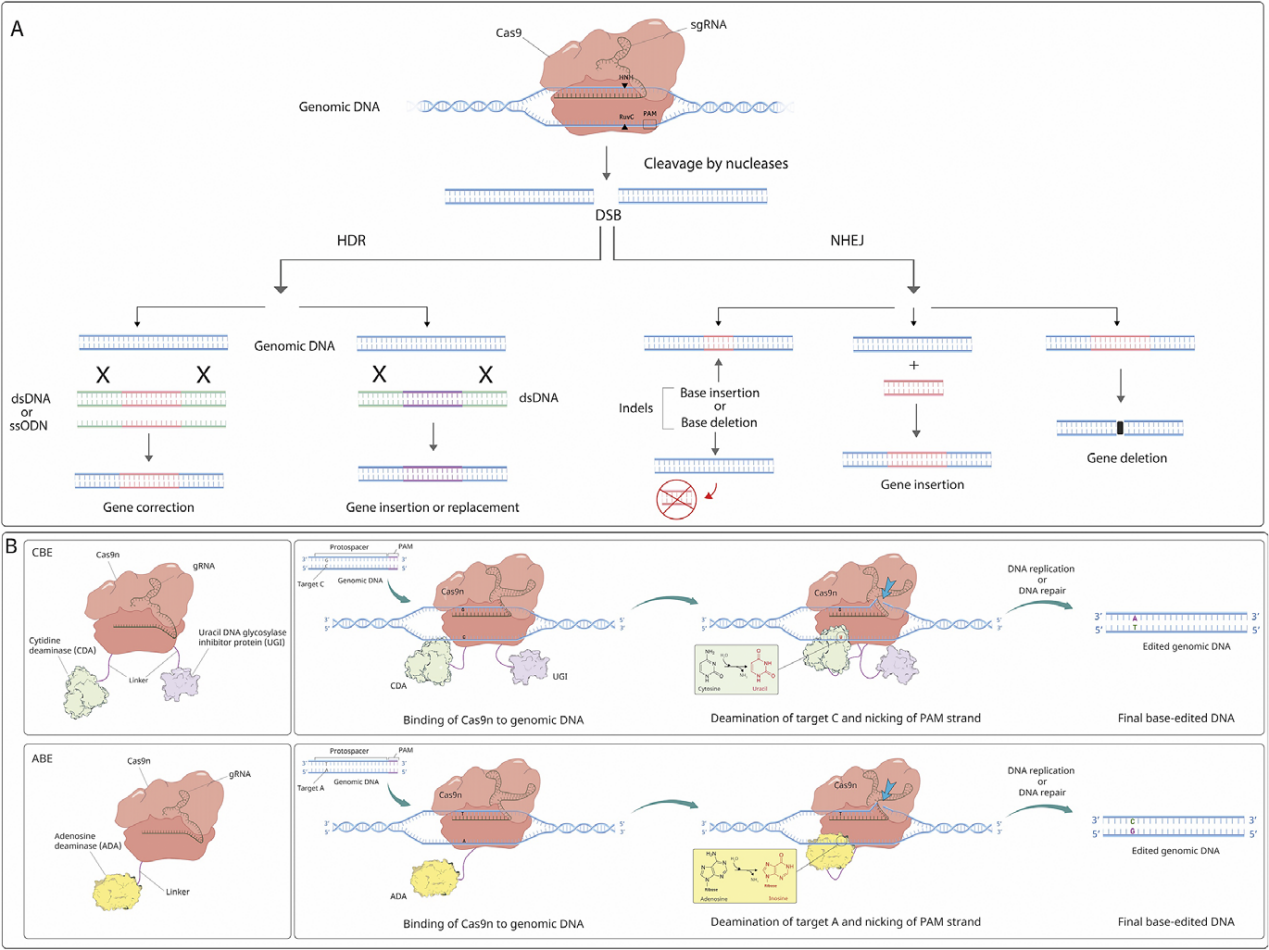
The potential applications of CRISPR-Cas systems for editing genomes and base editing technology (Refs 14, 15). Panel (a): CRISPR-Cas9 functions via a guide RNA molecule to target specific DNA sequences and a Cas9 protein to cleave the DNA at those target sites. This process allows for precise genome editing by either inducing DNA repair mechanisms to create mutations or by facilitating the insertion of new genetic material at the targeted location. Genome modification through CRISPR-Cas systems relies on the two primary pathways for repairing double-strand breaks (DSBs). Indel mutations and gene deletions result from the predominant nonhomologous end-joining (NHEJ) repair pathway. On the other hand, gene insertion, correction, and replacement occur through the homology-directed repair (HDR) pathway, utilising a DNA donor template. Panel (b): Base Editing Technology. The mechanism of the Cytosine Base Editor (CBE) is outlined, with key components labelled in text boxes. In the presence of the optional uracil glycosylase inhibitor (UGI), the U•G intermediate is safeguarded against excision by uracil DNA glycosylase (UDG), enhancing the efficiency of the final base-edited DNA outcome. The nickase version of Cas9 (Cas9n) induces a nick on the top strand (indicated by the blue arrow), while the cytidine deaminase transforms cytosine into uracil. The comprehensive conversion of a C•G to T•A base pair is accomplished through the specified steps. The mechanism of Adenine Base Editor (ABE) mirrors that of CBE, with the distinction that the UGI domain is not included in the ABE architecture. ABE-mediated editing leads to the conversion of an A•T to G•C base pair through an inosine-containing intermediate. Key elements include guide RNA (gRNA), protospacer adjacent motif (PAM), target A (desired base substrate for ABE), and target C (desired base substrate for CBE). The PAM sequence is representatively shown as 3 bp. dsDNA: double-stranded DNA, ssODN: single-stranded oligodeoxynucleotide.

CRISPR-Cas systems are revolutionary gene-editing tools that utilise a natural defence mechanism found in bacteria to precisely target and edit specific DNA sequences (Figure 2a) (Refs 11, 12). The Cas9 protein is guided to the desired location in the DNA by a small RNA molecule called guide RNA (gRNA) complementary to the specific DNA sequence to be edited. The gRNA is composed of two components: a CRISPR RNA (crRNA), which is responsible for recognising and binding to the target DNA sequence, and a trans-activating RNA (tracrRNA), which is essential for crRNA maturation and association with the Cas9 enzyme. A chimeric single guide RNA (sgRNA) synthetically designed to perform both these functions allows an equally functioning two-component system and facilitates its use in biotechnology (Ref 13).

*Mechanism.* The sequence to be edited by the CRISPR/Cas9 system must be adjacent to a short DNA sequence called Protospacer Adjacent Motif (PAM), which is necessary for Cas9 to recognise the target site. Once the sgRNA complexes with Cas9, the endonuclease adopts an active conformation that searches for the appropriate PAM sequence. Upon binding the PAM, local DNA melting is triggered downstream of the PAM, followed by the strand invasion of the sgRNA to test the potential DNA target for complementarity (Refs 16, 17). When adequate complementarity is detected between the sgRNA and the target site, the Cas9 enzyme will cleave both DNA strands at precise locations within the target sequence using its two active domains, HNH and RuvC, which act as molecular scissors. This results in a DSB in the DNA molecule. The therapeutic potential of CRISPR/Cas9 lies in its ability to induce such DSBs at specific genomic loci, prompting the cell to repair these breaks through endogenous DNA repair pathways. However, the inherent complexity and variability of these repair mechanisms pose significant challenges to the related therapeutic applications.

*DNA repair pathways induced by CRISPR-mediated DNA cleavage.* The two main pathways for DNA repair following the introduction of DSBs are non-homologous end joining (NHEJ) and homology-directed repair (HDR) (Figure 2a). NHEJ, which operates with high efficiency, involves direct ligation of the broken DNA ends back together via a process that is prone to errors, often resulting in small insertions or deletions (indels) at the site of the cut. These indels can disrupt the target gene function, leading to gene knockout. While these indels can be advantageous for gene disruption, they pose a challenge for precise gene editing due to unpredictable genomic consequences that complicate therapeutic outcomes (Ref 18). In the more accurate HDR pathway, the cell uses a template DNA molecule to perform a high-precision DNA repair. This allows the introduction of specific genetic modifications at the target site, such as gene knock-ins or precise nucleotide substitutions. This mechanism is highly suitable for applications ranging from basic research approaches to potential therapeutic interventions for genetic diseases (Ref 19). However, HDR is inherently less efficient than NHEJ and is known to occur only in the late S and G2 phases of the cell cycle. This limitation reduces the success of HDR-mediated edits in non-dividing or slowly dividing cells, such as neurones or cardiomyocytes, which are among the frequent targets in many therapeutic contexts (Ref 20). Other DNA repair pathways, such as base excision repair (BER) and mismatch repair (MMR), resolve perturbations induced by base editing, whereas those induced by prime editing (PE) are resolved by flap excision, thoroughly reviewed elsewhere (Ref 21). The efficiency and preference of DNA repair pathways can vary significantly between cell types. While the NHEJ pathway is the predominant DNA repair pathway in somatic cells, embryonic stem cells prefer the efficient HDR pathway (Ref 22).

*Base editing as a precise gene-editing technology.* Base editing is a modification of the traditional CRISPR-Cas9 system that is already being used in many clinical trials; it allows for precise and efficient editing of single nucleotides (adenine and cytosine) (Figure 2b) (Ref 23). This technique is useful for correcting point mutations or introducing specific nucleotide changes. Base editors are chimeric proteins consisting of a DNA-targeting module fused to a single-stranded DNA-modifying enzyme, such as cytidine deaminase or adenine deaminase, capable of directly converting one DNA base to a specific other (Refs 24, 25). A guide RNA (gRNA) is designed to direct the enzyme complex to the desired genomic location.

During the base editing process, the complex scans along the DNA for the target base after binding to the correct genomic location. When located, the deaminase enzyme within the base editor chemically modifies the target base without disrupting the DNA backbone. Cytidine deaminase base editors (CBEs) convert cytosine (C) to uracil (U), and adenine deaminase base editors (ABEs) convert adenine (A) to inosine (I). Following this step, the cell’s natural DNA repair machinery recognises the altered base and attempts to repair it. No DSBs, and thus no DSB-associated byproducts, are normally created (Ref 26). The base-modification enzyme in these systems operates on single-stranded DNA (ssDNA) but not double-stranded DNA (dsDNA). Upon binding to the target DNA region, base pairing between the gRNA and the target strand triggers displacement of a small segment of ssDNA in an R-loop, the DNA bases within which are modified. For improved efficiency in eukaryotic cells, the catalytically inactive nuclease also generates a nick in the non-edited strand, thus inducing repair with the edited strand taken as a template (Ref 26). Base editing offers several advantages over traditional CRISPR-Cas9 editing, including higher precision and reduced risk of off-target effects. Yet it is limited to converting specific types of DNA bases to others, but not to insert or delete longer stretches of DNA, though recent reports specify novel base editor types, including a dual base-editor system for combinatorial editing (Refs 27, 28). Another modification of the traditional CRISPR-Cas9 system is prime editing, which does not require dsDNA breaks as in base editing while having the further potential of making any substitution, small insertion and small deletion in DNA. This technology is yet in its infancy in clinical trials and is discussed in the future prospects section.

*Gene editing technologies compared.* Several targeted platform approaches focus on the development of novel treatment modalities for conditions such as immune system disorders, cardiovascular, metabolic and neurodegenerative diseases, viral infections, muscular dystrophy, haemophilia and T cell-based immunotherapies against cancer (Ref 1). CRISPR has gradually become a leading gene-editing technology, outperforming earlier approaches in key aspects like precision, efficiency, versatility and scalability. While ZFNs and TALENs both rely on protein-DNA interactions for target recognition, the sequence-specific cleavage in the CRISPR/Cas system is provided by the highly-specific RNA–DNA recognition via a gRNA, which can be synthesised or modified quickly and cost-effectively to target different sequences. In contrast, the protein engineering process for ZFNs and TALENs is labour-intensive and time-consuming, which can limit efficiency (Ref 29). Yet CRISPR/Cas technologies are still associated with a considerable level of off-target effects. These effects arise when the Cas enzyme functions on untargeted genomic sites, which may lead to several adverse outcomes. Since up to 3 mismatches between sgRNA and the genomic DNA can be tolerated by Cas9, the off-target regions are often considered sgRNA-dependent, although sgRNA-independent off-target effects are also known to occur (Ref 18). Overall, although off-target editing remains a concern with CRISPR/Cas systems, it is generally considered easier to mitigate than with ZFNs and TALENs. CRISPR technologies also stand out in their versatility and adaptability for different purposes, such as epigenome editing and transcriptional regulation, as well as multiplex genome engineering (Refs 30, 31).

**Figure 3.**
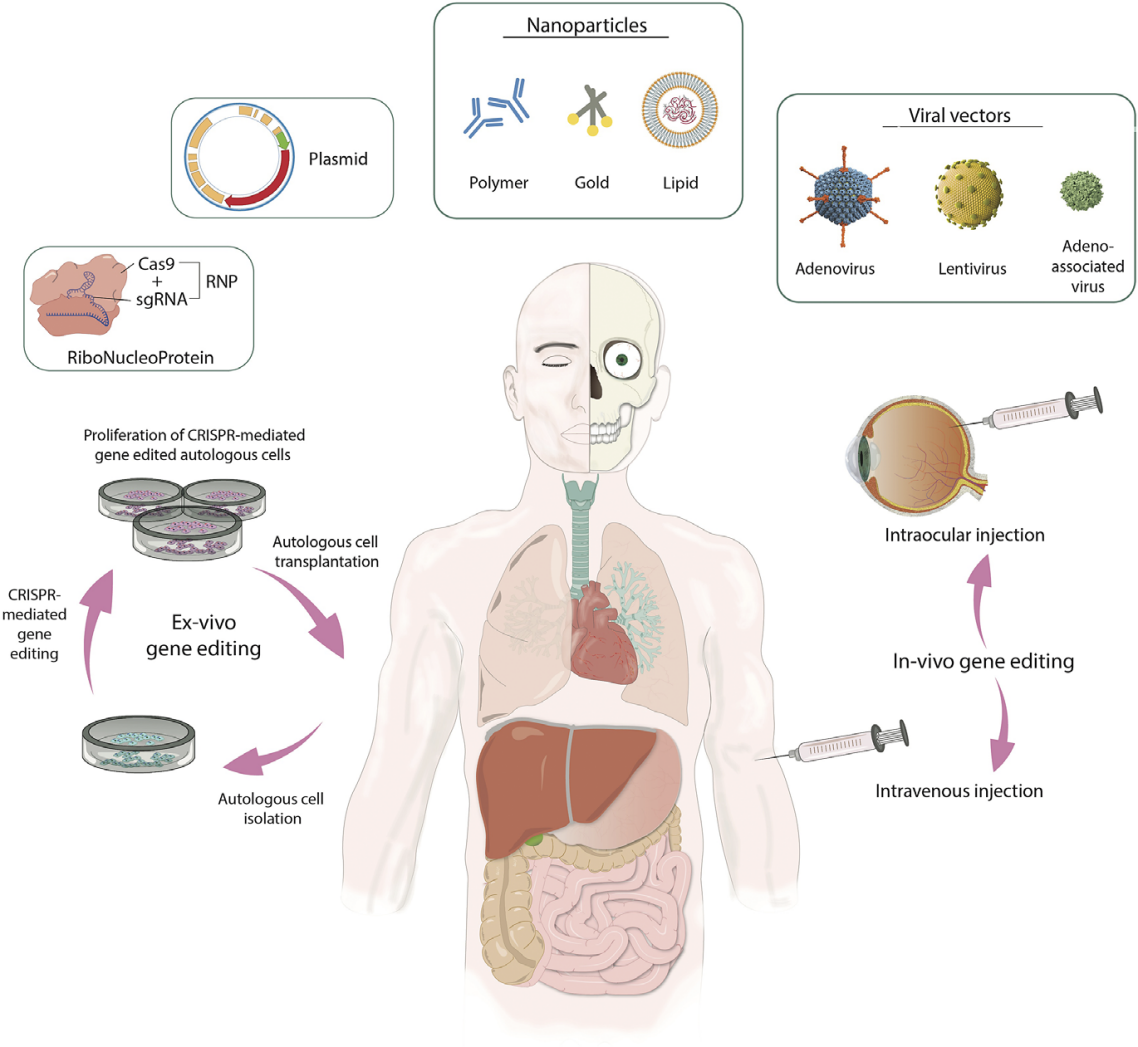
Strategies for gene modification therapies in humans. This figure illustrates two key approaches for therapeutic gene editing, as *ex vivo* and *in vivo. Ex vivo* gene editing involves the isolation and modification of patient cells using CRISPR/vector technology within a controlled *in vitro* environment. The genetically modified cells undergo proliferation before being transplanted back into the patient. In contrast, *in vivo* gene editing directly administers therapeutic genes using viral or non-viral vectors through intravenous or intraocular injection. The depicted gene editing methods include ribonucleoprotein (RNP), non-viral vectors (nanoparticles and plasmids), and viral vectors (adenovirus, lentivirus, and adeno-associated virus), showcasing the diverse strategies employed in the pursuit of targeted gene modifications.

The transition from the experimental applications of CRISPR towards clinical trials marks a significant milestone in genetic medicine (Ref 32). Preclinical studies often conducted in animal models provided crucial insights into the safety, efficacy and delivery methods of CRISPR therapies. This resulted in the CRISPR technology rapidly progressing toward therapeutic applications (Refs 33, 34). *In vivo* delivery systems used in preclinical and clinical CRISPR/Cas9 approaches are thoroughly reviewed in several highly informative reviews (Figure 3) (Refs 35–37). The potential of CRISPR technologies to address a wide range of genetic disorders is referred to in this review with a particular focus on clinical applications but also delving into some mechanistic insights and preclinical relevance for the interest of basic scientists, clinicians and other relevant professionals. The current challenges, possible solutions, and the need for rigorous evaluation in many aspects are also highlighted, along with ethical considerations.

## Clinical applications of CRISPR technologies

### Gene editing for patients with beta-haemoglobinopathies

Beta-haemoglobinopathies, the most common of which are sickle cell disease (SCD) and β-thalassaemia (BT), represent a collection of inherited monogenic recessive disorders characterised by faulty or reduced production of beta-globin chains, respectively. These conditions are associated with significant morbidity and mortality rates and are notably prevalent in the Mediterranean populations, Southern and Southeastern Asia, the Middle East, Africa and the Pacific Islands. They stand out as the most prevalent genetic disorders worldwide, with an estimated annual birth incidence surpassing 300,000 children.

In SCD, a single base substitution in the β-globin chain results in a missense mutation, replacing glutamic acid with valine at the sixth amino acid position. This alteration prompts the sickle haemoglobin to polymerise, distorting red blood cells into the characteristic sickle shape. These misshapen cells can block small blood vessels, resulting in compromised oxygen delivery to tissues and consequential complications such as pain crises, breathing difficulties and organ damage. On the other hand, BT is associated with inadequate β-globin production, which leads to an excess of unpaired α-globin chains precipitating in erythroid precursors. Thus maturation is impaired, resulting in precursor cell death and ineffective erythrocyte production. The ensuing significant anaemia and expansion of erythroid precursors contribute to secondary issues in bones and other organs. Despite available treatments for both diseases, severe symptoms and complications may still exist even with intervention. Bone marrow transplantation is a potential cure that relies on finding a healthy, compatible donor, limiting its feasibility to only a fraction of patients. It is also associated with risks of transplant-related mortality, graft-versus-host disease (GVHD) and graft rejection (Ref 38). The majority of individuals with SCD or BT depend on regular frequent, often lifelong blood transfusions as a critical part of their management. This is typically combined with iron chelation therapy (ICT) to prevent excess iron from transfused red blood cells from accumulating in the body and damaging vital organs such as the heart and liver (Refs 39, 40). Among the major limitations of these approaches are the scarcity of blood products, which leads to a lack of adequate and safe blood transfusions, as well as low accessibility to ICT, treatment toxicity, adverse events (including alloimmunisation, transfusion-related reactions and infections) and high costs (Ref 41). These limitations underscore the need for curative therapies, including foetal haemoglobin (HbF) induction via gene editing, as a feasible and efficient approach with the potential to provide long-term solutions for beta-haemoglobinopathies.

The transition from foetal to adult haemoglobin and suppression of HbF during human development have long captured interest. HbF is a type of haemoglobin produced by feotuses in the womb but absent in children and adults, remaining unaffected by sickle cell mutation. Clinical observations have consistently indicated that enhanced HbF production mitigates the severity of SCD and BT. In individuals with SCD, symptoms typically emerge in infancy as HbF levels naturally decline. Accordingly, asymptomatic SCD until after infancy was attributed to elevated HbF levels initially based on clinical observations (Ref 42). This concept gained support from the study of rare patients with compound heterozygosity for SCD and hereditary persistence of HbF mutations, who exhibited predominantly asymptomatic profiles. Subsequent larger epidemiological studies in SCD confirmed that elevated HbF levels substantially and quantitatively alleviate clinical severity while reducing mortality (Refs 43–46). Similar patterns emerged in patients with BT. Observations in rare BT cases with increased HbF production revealed a milder clinical course; infants manifested symptoms only after the decline in HbF expression in the months following birth (Refs 43, 47). Larger epidemiological studies within thalassaemia populations consistently confirmed these findings (Refs 48–50).

**Figure 4.**
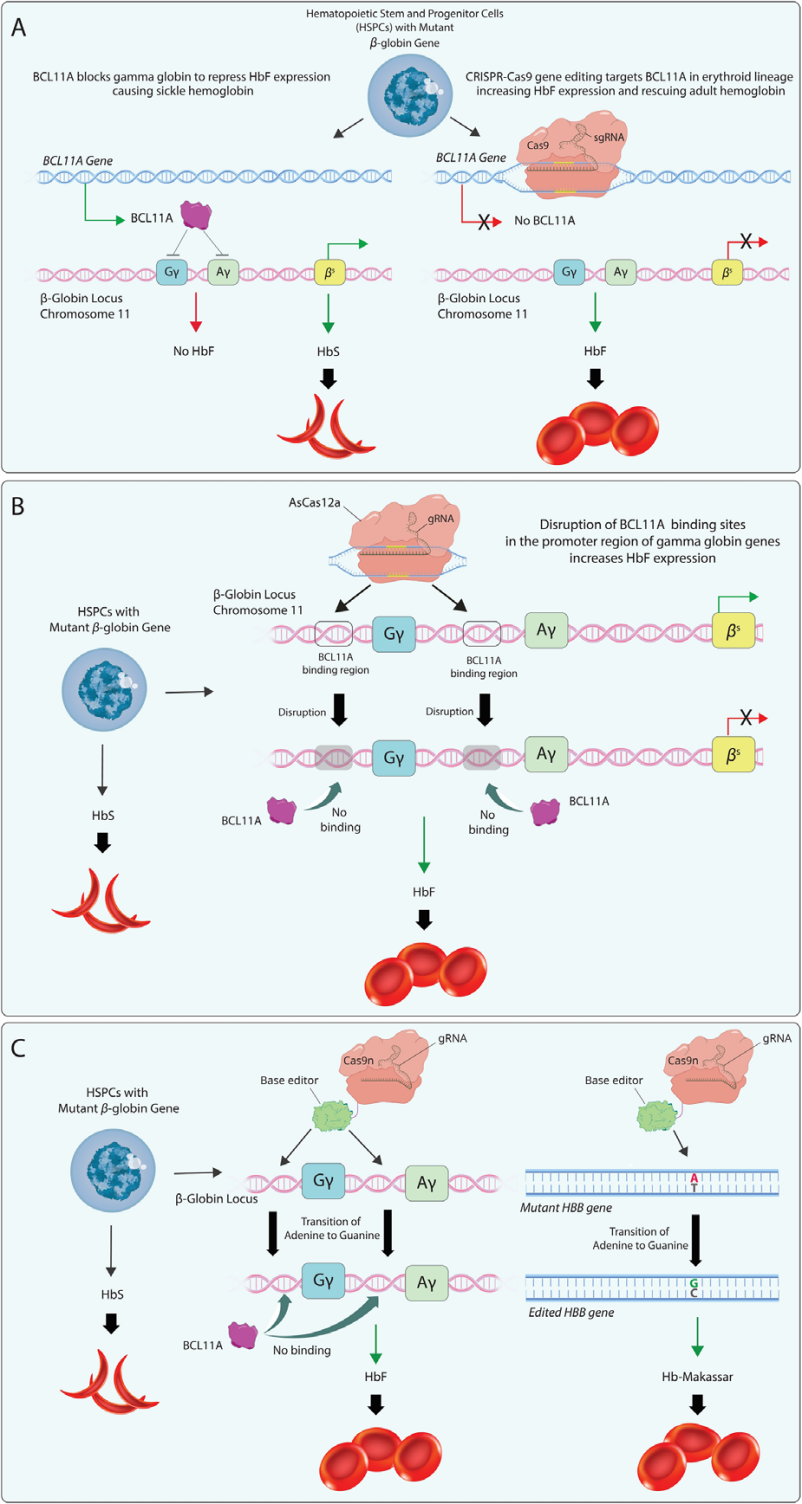
CRISPR-based gene editing strategies to correct beta haemoglobinopathies such as sickle cell disease (SCD) and beta-thalassaemia (BT). CASGEVY, developed by Vertex and CRISPR Therapeutics, entails the genetic modification of a patient’s own HSPCs via CRISPR/Cas9 and SPY101 single guide RNA (Ref 54). This modification aims to disrupt the GATA1 transcription factor binding domain of the B-cell lymphoma/leukaemia 11A (BCL11A) gene erythroid enhancer through ex vivo editing. BCL11A, a known suppressor of foetal haemoglobin (HbF) expression, presents a target for intervention. Consequently, this disruption leads to a significantly increased HbF expression, effectively correcting the deficient production of adult beta haemoglobin (Panel A). Another notable approach (EDIT-301) developed by Editas Medicine involves targeting the promoters of the γ-globin genes [HBG1 (Aγ) / HBG2 (Gγ)], introducing distinct sequence alterations to interfere with BCL11A binding sites, leading to enhanced production of HbF (Panel B) (Ref 55). This alteration is accomplished by employing the AsCas12a protein, which is well-known for its superior efficiency and specificity in gene editing. The CRISPR base editors are also the subject of intense interest, with two primary methods developed by the BEAM Therapeutics for addressing haemoglobinopathies (Panel C). The first one, BEAM-101, involves performing an A-G transition in the BCL11A binding regions located in the promoter regions of gamma-globin genes to prevent the binding of BCLA11A, thereby increasing gamma-globin expression (Ref 56). The preclinical BEAM-102, the latter of the two, involves converting adenine to guanine at the specific point in the mutant beta-globin gene responsible for sickle cell formation (Ref 57). Due to this process, the haemoglobin produced, known as Haemoglobin Makassar, inhibits the formation of sickle cells. Other clinical trials, such as those involving the replacement of the mutated beta-globin gene through CRISPR-Cas9 knock-in (CRISPR_SCD001) and the correction of mutations in HBB to restore normal haemoglobin expression (GPH101), are omitted for clarity.

The clinical induction of HbF production thus held great promise in alleviating the severe symptoms associated with SCD and BT (Ref 51). Although non-specific pharmacological inducers displayed some success at inducing HbF, more effective and targeted approaches were required in the clinical setting (Ref 52). The most advanced approach to filling this gap followed an innovative route by elevating HbF levels via genome engineering rather than restoring healthy adult haemoglobin (Figure 4). The initial phase of treatment involves the collection of CD34+ haematopoietic stem cells (HSCs) directly from the patient’s bloodstream, followed by genome modification to activate the HbF gene. The patient then receives chemotherapy to eliminate ailment-triggering blood stem cells, making way for the edited cells. Lastly, the genome-edited stem cells are reintroduced into the patient’s bloodstream through intravenous (IV) administration. The goal is for these edited cells to establish themselves in the bone marrow and create a fresh population of blood stem cells that exclusively produce HbF-expressing erythrocytes. This *ex vivo* genome editing approach ensures that the genome-editing tools specifically interact with the intended target cells and mitigates the risk of persistent CRISPR components in the body, thus reducing the chances of unintended edits or immune reactions (Ref 53).

The initial CRISPR-based clinical trial entailing the use of CRISPR to reawaken HbF production for SCD and transfusion-dependent β-thalassaemia (TDT) received support from Vertex Pharmaceuticals (Boston, Massachusetts) and CRISPR Therapeutics (Zug, Switzerland) (Ref 58). In this strategy, the erythroid-specific enhancer region of the *BCL11A* gene, which prevents the production of HbF, is targeted and cut in both strands by Cas9 (Figure 4a). Once disrupted, this gene can no longer block HbF production, allowing it to display a therapeutic effect by boosting oxygen supply to tissues.

Despite not directly addressing the mutations responsible for SCD or BT, this treatment modality proved functional as a practical cure for both conditions. In November 2023, the U.K. Medicines and Healthcare Products Regulatory Agency (MHRA) approved this one-time IV treatment of CRISPR-edited cellular therapy under the commercial name of Casgevy for conditional marketing authorisation. The treatment is aimed for use in SCD patients 12 years of age and older with recurrent vaso-occlusive crises or TDT patients eligible for HSC transplantation, for whom an HLA-matched HSC donor is not available. In the trial for SCD, 29 out of 45 participants were followed long enough to announce reliable results; 28 of these patients no longer suffered from the vaso-occlusive crises characteristic of the disease at least 1 year following treatment. The same regimen was tested for TDT, and out of the 54 people who received the treatment, 42 participated for sufficient duration to draw reliable conclusions. Among these patients, transfusions were unnecessary for at least 1 year for 39 individuals, while three patients experienced a 70% reduction in transfusion requirement (Ref 59).

Casgevy received FDA approval for SCD in December 2023, followed by European Medicines Agency (EMA) approval in February 2024. According to the FDA, it is the first FDA-approved treatment to employ a novel genome editing technology, marking a groundbreaking advancement in the field of gene therapy. The results of this single-arm multicenter trial of safety and efficacy testing in adolescent and adult SCD patients were announced by the agency as follows: Casgevy treatment was administered to 44 patients, and out of the 31 individuals who were monitored for an adequate period to assess their condition, 29 achieved relief from vaso-occlusive crises lasting at least 12 consecutive months. The FDA’s report also stated that there were no instances of graft failure or rejection. Low platelet and leukocyte levels, nausea, abdominal pain, mouth sores, musculoskeletal pain, headache, itching and febrile neutropenia were presented as the most common side effects.

Intriguingly, Editas Medicine, Inc. is currently conducting two phase 1/2 trials for individuals with severe SCD (RUBY trial) and TDT (EdiTHAL trial), employing a CRISPR system featuring AsCas12a protein (EDIT-301: renizgamglogene autogedtemcel: reni-cel) (Figure 4b) (Ref 60). The method involves genomic modification of the γ-globin gene promoters [*HBG1* (Aγ) / *HBG2* (Gγ)] to interfere with the *BCL11A* binding sites to reactivate γ-globin expression, thus increasing HbF production in autologous HSCs. The study marks the first instance of Cas12 being utilised in a clinical trial. A detailed update by the company on 11 December 2023, included the safety and efficacy data in 11 patients enrolled in RUBY and 6 in EdiTHAL. All treated patients in the RUBY trial were reported to be free of vaso-occlusive crises since the infusion, which induced an early and substantial increase in total and foetal haemoglobin. Normal haemoglobin levels and an HbF level of >40% were reported in 6 patients throughout 5–18 months of follow-up. A similar early and substantial rise in the total and foetal haemoglobin levels was also evident in the efficacy results reported for the EdiTHAL trial; importantly, the total haemoglobin increased above the transfusion dependence threshold (9 g/dL). As of October 2024, the company announced that 28 patients received the drug in the RUBY trial, which was well tolerated, at a median of 9.5 months follow-up. Eleven patients had over one year of follow-up. Twenty-seven of the patients were reported to be free of vaso-occlusive events, with early normalisation of total haemoglobin. Mean total haemoglobin increased from 9.8 g/dL at baseline to 13.8 g/dL at month 6 (n = 18) (Ref 61). New safety and efficacy data for the EdiTHAL trial presented at the 66th American Society of Hematology (ASH) Annual Meeting and Exposition revealed that the 7 patients, who were at a median (range) of 10.5 (6.3–15.1) months post-infusion with two patients having over 1-year follow-up, had total haemoglobin levels remaining above the transfusion-independence threshold of 9.0 g/dL. This level increased to 12.5 (1.5) g/dL by month 6. Overall, all 7 patients were announced to be transfusion independent for a range of 5.8–14.5 months following the last red blood cell transfusion at 0.7–2.2 months post-reni-cel infusion. The company reveals the safety profile as consistent with myeloablative conditioning with busulfan, with no serious adverse effects reported related to the drug (Ref 62).

Furthermore, Beam Therapeutics initiated their phase 1/2 trial (BEACON) for a base editing therapy targeting severe SCD in the United States in November 2022, and the dosing of the first patient was announced in January 2024 (Ref 56). The therapeutic named BEAM-101 used in this trial involves an A-G transition in the *BCL11A* binding site within the promoter regions of the γ-globin genes, which the company claims to have several advantages over other therapeutics, as a ‘next-generation form of CRISPR’. The most significant benefit of the approach seems to lie in its action mechanism, excluding a double-strand cut in DNA but instead involving precise, single-letter changes mimicking single nucleotide polymorphisms involved in the hereditary persistence of foetal haemoglobin (HPFH). Undesired chromosomal abnormalities and genotoxic stress are claimed to be prevented via this modality. The company revealed clinical data from 7 patients in the 66th American Society of Hematology (ASH) Annual Meeting and Exposition in December 2024, stating that >60% HbF induction and <40% Haemoglobin S (HbS) reduction along with resolution of anaemia were achieved in all 7 patients (Ref 63).

Another base-editing strategy in SCD used a custom ABE (ABE8e-NRCH) that converts the sickle cell allele to the *HBB^G^* Makassar allele, a non-pathogenic variant reported in individuals living in the Makassar region of Indonesia (Ref 57). mRNA encoding the BE with a targeting gRNA was delivered *ex vivo* into haematopoietic stem and progenitor cells (HSPCs) from patients with SCD. The researchers reported an 80% conversion of *HBB^S^* to *HBB^G^.* Durable gene editing was evident with 68% frequency of *HBB^G^* and fivefold-decreased hypoxia-induced sickling of bone marrow reticulocytes 16 weeks following transplantation of the edited human HSPCs into immunodeficient mice, demonstrating preclinical therapeutic relevance. Beam Therapeutics is now testing the HbG-Makassar direct editing strategy via its preclinical drug BEAM-102 (Figure 4c).

One other advancement towards the treatment of SCD is the replacement of the mutated β-globin gene through CRISPR-Cas9 knock-in in a planned phase 1/2 trial in subjects ≥12 years old to 35 years old with SCD, via a single infusion of sickle allele-modified CD34+ HSPCs (CRISPR_SCD001). Also, nulabeglogene autogedtemcel, formerly known as GPH101, has been announced as the first CRISPR-based therapy candidate aiming to correct the *HBB* point mutation to restore normal haemoglobin expression. The phase 1/2 CEDAR trial was initiated to assess GPH101 regarding safety, efficacy and pharmacodynamics in adults and adolescents with severe SCD. In 2022, a single participant was dosed in a phase 1/2 trial, employing a combination of electroporation to deliver the CRISPR proteins into the cell and a viral vector to introduce a DNA ‘template’ for copying the new gene variant into the cell. In early January 2023, the company disclosed that the initial participant exhibited prolonged decreased blood cell counts (pancytopenia), necessitating continual blood transfusions and other therapies. Thus, discontinuance of the programme was announced in February 2023 to seek a partnership agreement for the external development of the drug (Ref 64).

Beta haemoglobinopathies are among the diseases that will benefit a great deal from gene editing approaches, as even partial correction of related mutations with a suitable strategy may provide adequate levels of functional haemoglobin production and mitigate disease severity. One of the primary limitations of the CRISPR-Cas9 HDR system for disease correction is its relatively low efficiency in quiescent cells and the formation of large unintended deletions and chromosome-level changes resulting from the DSBs. Additionally, indels in the coding region of the β-globin locus could result in severe β0-thalassaemia phenotypes. Disruption of HbF repressors or upregulation of HbF expression via the introduction of HPFH-like mutations through base editing approaches are attractive strategies to compensate for the deficient beta globin, along with those to correct beta-thalassaemia point mutations (Ref 65). Researchers point out that uncontrolled mixtures of Cas9-mediated indels and other challenges, such as an adaptive immune response against Cas9 protein and activation of the p53 pathway in human stem cells, may lead to a reduction in CRISPR/Cas9 editing efficiency in clinical applications, hindering HSPC proliferation and engraftment (Refs 66, 67). Base editing and PE techniques eliminate such consequences to a great extent, as strong alternative approaches that do not rely on DSBs like Cas9 nucleases (Ref 68).

### Rearming of T-Cells via gene editing against cancer

T cells are an important group of lymphocytes pivotal to the immune system, playing a key role in anticancer immunity. They navigate the body to eliminate foreign or harmful cells and recruit other immune cells for assistance. Their functions are mediated through diverse specialised T-cell receptors that distinguish between safe and threatening cells (Ref 69). Chimeric antigen receptor (CAR) T-cells are promising new genetically engineered cell-based drugs against cancer.

Many approaches involving CAR-T therapies are autologous, where T cells extracted from a patient’s blood are reinfused to the patient after being genetically modified and multiplied. It’s an effective yet costly and time-intensive treatment, with bottlenecks in the manufacturing process. Thus, a primary focus is the development of allogeneic CAR T-cells, sourced from a healthy donor and modified to specifically attack cancer cells while avoiding detection by the recipient’s immune system. These edited cells are subsequently multiplied into substantial quantities, enabling widespread administration to numerous recipients as needed. Reduced costs and shorter preparation times are major advantages of allogeneic products, as well as providing robust high-quality cells for on-demand cancer immunotherapy (Ref 70).

**Figure 5.**
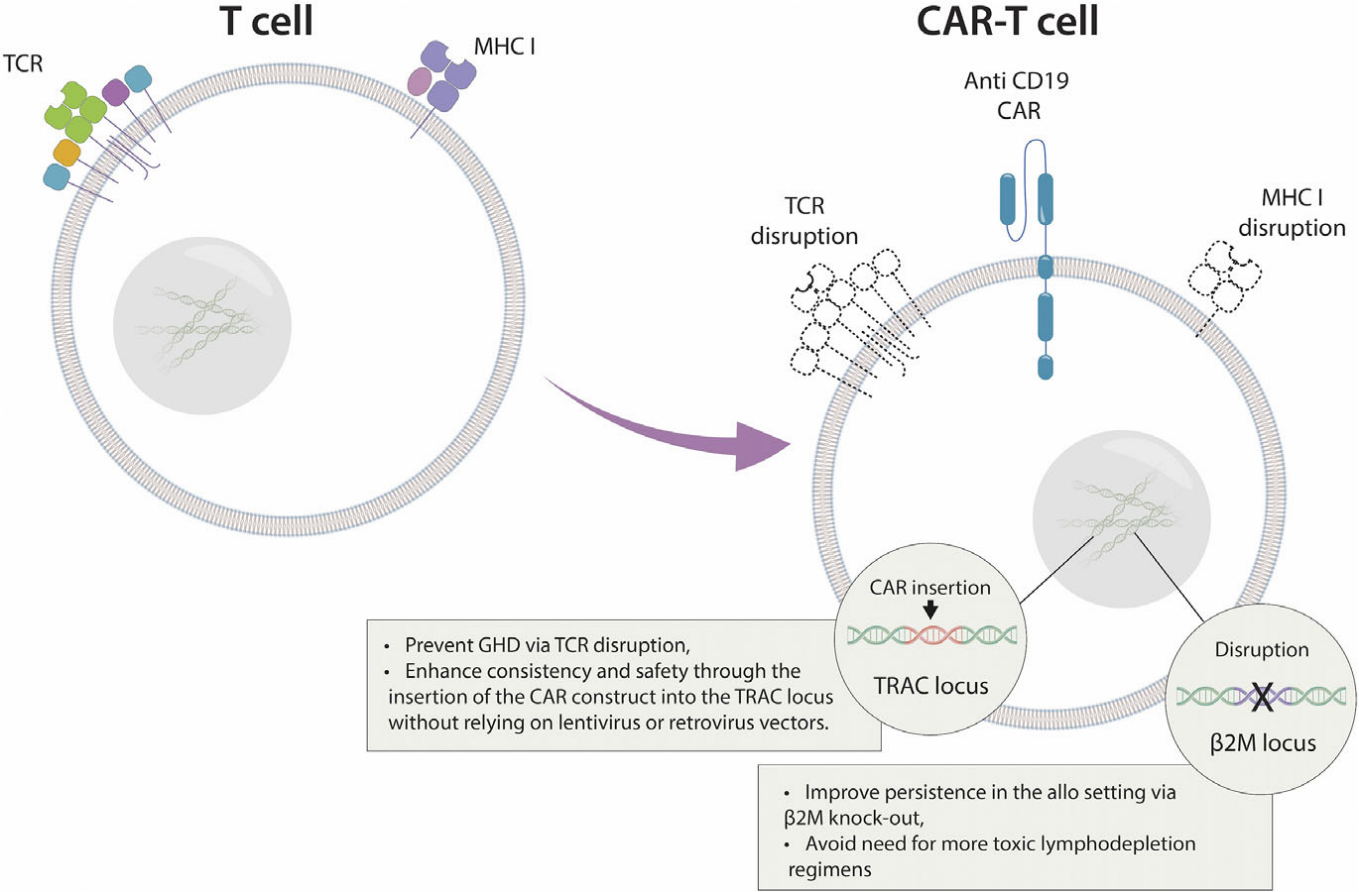
Distinctive design of allogeneic CAR T-cells modified using CRISPR technology. CTX110 is a chimeric antigen receptor T-cell (CAR-T) therapy developed by CRISPR Therapeutics (Ref 71). It is designed to target and treat cancers by modifying a patient’s T cells to recognise and attack cancer cells expressing the CD19 antigen. CTX110 uses CRISPR gene editing technology to precisely modify T cells to express a synthetic receptor (CAR) that targets CD19, allowing the modified T cells to recognise and destroy cancer cells expressing this antigen. CTX110 is currently being investigated in clinical trials for the treatment of various haematologic malignancies, including non-Hodgkin lymphoma and chronic lymphocytic leukaemia. CTX120 and CTX130 employ a similar CRISPR-edited allogeneic T cell framework, differing in their CAR targets and, in the case of CTX130, incorporating additional editing.

CRISPR Therapeutics is currently investigating the effects of allogeneic CRISPR-modified CAR-T cell variants (Figure 5). The company’s first allogeneic T-cell products, CTX110 (targeting CD19+ malignancies) and CTX130 (targeting CD70+ malignancies), were announced to have favourable results in B- and T-cell lymphoma and renal cell carcinoma (NCT04035434, NCT04502446, and NCT04438083, respectively). CD19 is a protein that is frequently present in leukaemia and lymphoma cells. CD70 is a protein commonly overexpressed in cancer cells of various solid and haematological origins. CTX130 was tested in relapsed/refractory T- or B-cell malignancies under the COBALT-LYM trial and relapsed/refractory renal cell carcinoma under the COBALT-RCC trial. The drug received FDA Orphan Drug and Regenerative Medicine Advanced Therapy (RMAT) designations. These two treatments were, however, also associated with T cell exhaustion leading to loss of response and reduced efficacy, particularly in high tumour burden patients. Thus new edits via CRISPR/Cas9 technology were included in the ‘next-generation’ CAR T cell programmes, applied under the names of CTX112 and CTX131. The company describes three distinct modifications in healthy donor T lymphocytes in preparation for these treatments. Firstly, aiming to block the host-versus-graft disease (HVGD), class 1 major histocompatibility complex (MHC I) is eliminated by knocking out the β2 M subunit (Ref 71). This increases persistence and the chance for durable remissions. Secondly, CRISPR/Cas9 eliminates the existing TCRs, aiming to reduce the risk for GVHD. Lastly, CRISPR/Cas9 technology is used to insert the CAR construct into the TCR alpha constant (*TRAC*) locus to improve safety and consistency.

Additional teams have achieved remarkable outcomes by targeting CD19 in the context of challenging and aggressive B-cell non-Hodgkin lymphomas. Caribou Biosciences applied a promising technology in their treatment approach; in addition to directing their cells toward CD19, they incorporated a second genetic alteration, a ‘knockout’ deactivating the programmed death-1 *(PD-1)* gene, often used by the cancer cells for their advantage to evade the immune system (Ref 72). This approach is designed to enhance antitumour activity by restricting premature CAR T-cell exhaustion. The strategy utilises Cas9 chRDNA guides to make the necessary edits. It is a technology defined by the company as a CRISPR hybrid RNA–DNA (chRDNA), aiming to improve CRISPR genome-editing precision through the highly reduced affinity of the chRDNA guide to the off-target sequences. Mismatches between the chRDNA guide and off-target sites significantly reduce the stable binding of the Cas complex, thereby hindering cleavage by the Cas nuclease. As of July 2023, Caribou Biosciences shared their long-term follow-up results for their product CB-010 under the ANTLER Phase 1 clinical trial (Ref 73). Notably, the treatment demonstrated a generally well-tolerated and safe profile. In this dose-escalation study involving 16 patients, a 94% overall response rate was reported, with 69% of the patients (11 of 16) displaying a complete response (CR). Seven of the 16 patients achieved CR for over 6 months, with the longest CR announced as 24 months. A related abstract presented for the 2024 ASCO Annual Meeting also stated a manageable safety profile and promising efficacy in patients with refractory/resistant B-NHL, with the dose escalation phase being completed (Ref 74). The company also pursues Phase 1 trials involving allogeneic anti-BMCA CAR-T cell therapy for relapsed or refractory multiple myeloma (CB-011) and allogeneic anti-CLL-1 CAR-T cell therapy against relapsed or refractory acute myeloid leukaemia (CB-012), where a Cas12a chRDNA genome-editing technology is used. CB-010 holds RMAT, Fast Track and Orphan Drug FDA Designations, whereas CB-011 and CB-012 hold Fast Track and Orphan Drug FDA Designations. Both trials are currently recruiting patients.

Intriguingly, in a recent strategy involving the generation of off-the-shelf allogeneic CAR T cells, lentiviral-mediated expression of a CAR targeting CD7 (CAR7) was obtained on healthy donor T cells, followed by base editing for the inactivation of three genes encoding the CD52 and CD7 receptors along with the β chain of the αβ T-cell receptor (Ref 75). These modifications were carried out to prevent lymphodepleting serum therapy, CAR7 T-cell fratricide, and GVHD, respectively. The safety of these edited T cells was investigated in 3 children with relapsed leukaemia. The first patient was a 13-year-old girl with relapsed T-cell ALL following allogeneic stem-cell transplantation. Molecular remission within 28 days was reported after the single-dose base-edited CAR7 treatment (BE-CAR7). A nonmyeloablative allogeneic stem-cell transplantation from the patient’s original donor followed this process, leading to ongoing leukemic remission. BE-CAR7 cells were effective in the other two patients in the same trial, although one developed progressive lung complications related to cytokine release syndrome along with fatal fungal complications. The third patient received allogeneic stem-cell transplantation during remission. These results indicated the anticipated risks related to immunotherapy-related complications in this phase I study, where cytokine release syndrome, multilineage cytopenia, and also opportunistic infections were reported as serious adverse effects.

In another recent phase I trial, a simultaneous knockout of the endogenous *TRAC* (encoding TCRα) and *TRBC* (encoding TCRβ) genes was performed via CRISPR-Cas9 genome editing, along with two chains of a neoantigen-specific TCR (neoTCR) acquired from patients’ circulating T cells inserted into the *TRAC* locus. The trial, sponsored by PACT Pharma, involved 16 patients with different refractory metastatic solid cancers, including melanoma, urothelial carcinoma, head and neck squamous cell carcinoma, non-small cell lung carcinoma and colorectal, ovarian, prostate and hormone receptor-positive and triple-negative breast cancers. This approach is unique in assessing the genetic makeup of an individual’s tumour and then utilising CRISPR technology to customise the patient’s T cells to specifically target the individual disease. Each participant received up to three distinct engineered T cells. Five patients displayed stable disease; a high percentage of neoTCR transgenic T cells were reported in the periphery, and there was a decrease in some target lesions; thus, the therapy was considered likely to have had an effect. All patients were reported to display the expected side effects associated with lymphodepleting chemotherapy (Ref 76).

Overall, CAR-T cell therapy emerged as a strong treatment strategy for malignant tumours. Yet the survival and persistence of CAR T-cells are often impaired due to their terminally differentiated phenotype and exhausted status. CRISPR/Cas9 technology has been used in various trials to reduce exhaustion, generate a memory phenotype, and look for new targets to improve anti-cancer potential, providing an effective strategy to efficiently promote the proliferation and persistence of CAR T-cells *in vivo* (Ref 77). Yet challenges such as off-target effects and Cas9 protein-mediated immunogenicity limit the application of the CRISPR/Cas system to CAR T-cells. Rational designs of sgRNAs by bioinformatics tools, use of alternative Cas nucleases and adjustment of delivery systems are a few measures to avoid the off-target effects. Strategies such as epitope masking are among the solutions to the Cas9 protein-related immunogenicity in the *in vivo* CRISPR/Cas9 editing (Ref 78). Overall, CAR T-cell therapies face other challenges such as the emergence of T-cell malignancies, including CAR-positive lymphoma. T-cell lymphomas are especially notable in this clinical context due to concerns that CAR T-cell vector integration may contribute to cancer development. Researchers emphasise the infrequent occurrence of second tumours in CAR T-cell applications, while still acknowledging it as a significant concern (Refs 79–82). Incorporating CRISPR into these therapies may improve the approach by utilising a more precise strategy than conventional vector integration.

Patients undergoing CAR T-cell therapy often encounter cytokine release syndrome (CRS), a severe adverse event triggered by systemic levels of pro-inflammatory cytokines such as interleukin-6 (IL-6), tumour necrosis factor (TNF), and interferon-gamma (IFN-γ) (Ref 83). The condition is characterised by life-threatening risks such as severe fever, hypoxia, and organ damage. CAR’s engagement with its target antigen, initial cytokine release from activated CAR T-cells, and subsequent activation of bystander immune cells contribute to the pathophysiology of CRS. This leads to the release of a broad spectrum of cytokines from both CAR T-cells and native immune cells, accompanied by the expansion of CAR T-cells. CRISPR-Cas9 editing may also be very useful in addressing this problem, as in the approach where the technology was used to modify CAR T-cells with a GM-CSF genetic knockout, decreasing the production of proinflammatory cytokines and chemokines (Ref 84).

### Genetic engineering of photoreceptors for genetic blindness

Leber Congenital Amaurosis (LCA) is among the earliest and most severe forms of inherited retinal dystrophies (IRDs), responsible for 20% of early childhood blindness (Ref 85). The disease is characterised by degeneration and/or dysfunction of photoreceptors and eventual death of retinal cells. The most prevalent form of the disease, LCA10, occurs due to mutations in the centrosomal protein of 290 kDa (*CEP290)* gene. The CEP290 protein plays an important role in cilium assembly and ciliary protein trafficking, localised in the connecting cilium as a multi-protein complex required for structural and functional integrity. Thus, when individuals with LCA10 are exposed to light, these compromised cells are unable to effectively transmit all the necessary signals to the brain, resulting in loss of vision. The CRISPR-based approach for LCA10 treatment aims to address this issue by modifying the defective photoreceptor gene, prompting it to produce a complete and functional protein instead of the defective, truncated version. The goal is to edit a sufficient number of cells to generate healthy protein for the patients to regain their lost vision.

**Figure 6.**
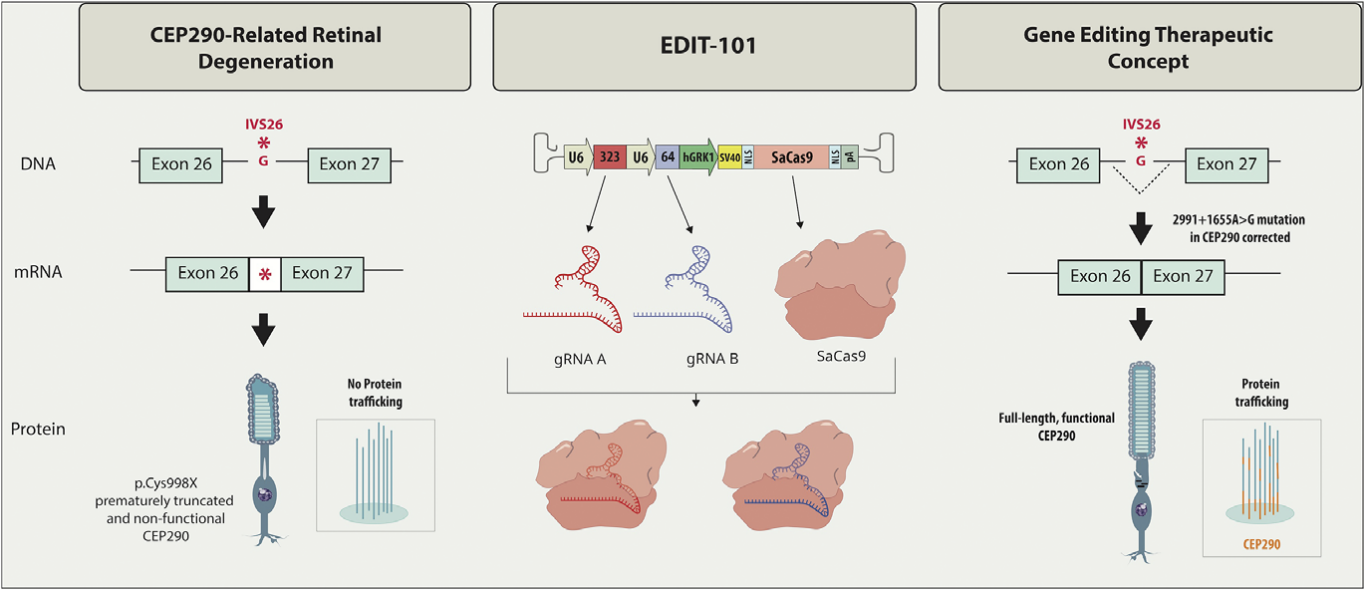
A gene-editing approach for genetic blindness. EDIT-101 is a novel gene therapy developed by Editas Medicine, aimed at treating Leber congenital amaurosis 10 (LCA10), a rare genetic form of blindness (Ref 88). It utilises CRISPR-Cas9 gene editing technology to correct mutations in the *CEP290* gene, responsible for the LCA10 phenotype. An AAV5 vector was used to deliver the *Staphylococcus aureus* Cas9 (SaCas9) and CEP290-specific guide RNAs (gRNAs) to photoreceptor cells by subretinal injection. By targeting and repairing the faulty genetic sequence, EDIT-101 aims to restore vision in affected individuals. The therapy is administered through intraocular injection, directly into the eye, allowing it to target retinal cells. U6: human U6 polymerase III promoter; 323: gRNA; CEP290–323; 64: gRNA CEP290–64; hGRK1: human G protein-coupled receptor kinase 1 promoter; SV40 SD/SA: simian virus 40-splice donor and splice acceptor containing intronic sequence.

EDIT-101 represents an experimental medicine based on CRISPR/Cas9 editing, aimed at eliminating the abnormal splice donor site induced by the c.2991+1655A>G IVS26 mutation in *CEP290* (Figure 6) (Ref 86). To reinstate normal CEP290 expression, an upstream sgRNA guides the initial Cas9 cleavage to a location preceding the IVS26 mutation, while a downstream sgRNA directs the second Cas9 cleavage to a site situated beyond the mutation. The resulting cleavage ends undergo direct ligation through the NHEJ process, and thus the intronic fragment flanking the IVS26 mutation is removed (Ref 87). The mRNA processing machinery subsequently eliminates the truncated intron 26 during RNA splicing. EDIT-101 is delivered through a subretinal injection to precisely target and convey the gene editing machinery directly to photoreceptor cells (Ref 88).

The first *in vivo* CRISPR therapy trial, conducted in the United States and sponsored by Editas Medicine, targeted the LCA10 (Ref 86). Commencing in March 2020 with the first patient receiving treatment, successive dosing of limited cohorts was extended until July 2022. Editas initially administered low-dose treatments to adult cohorts before progressing to high-dose adult cohorts and a paediatric cohort. This sequential approach aimed to mitigate potential hazardous side effects throughout the trial, especially concerning the paediatric group. The subretinal administration involved treating one eye, while the other eye served as a control for assessing vision in the treated eye. According to Editas’ official statements, no severe adverse events or dose-limiting toxicities surfaced during the trial. Evaluating treatment efficacy posed a greater challenge than ensuring safety in these cases. Directly gauging the percentage of edited cells or detecting unintended edits in participants proved difficult. Due to the substantial reduction in vision, conventional line-by-line letter reading tests were impractical. Instead, alternative assessments such as mobility tests (e.g., navigating obstacles) and light detection capabilities were employed (Refs 53, 86).

During their phase 1/2 trial named BRILLIANCE for testing EDIT-101, Editas disclosed that merely 3 among 14 patients had shown ‘clinically meaningful’ improvements in their vision by November 2022 (Ref 86). Notably, two of these responsive individuals harboured mutations in both copies of the pertinent gene, hinting at the potential effectiveness of the treatment within this specific subset of the LCA10 population. This particular subgroup, existing within an already rare condition, comprises only approximately 300 individuals in the US. Owing to the exceedingly limited patient pool for this costly drug, Editas paused enrolment in the BRILLIANCE trial, yet keeping the possibility of resuming the efforts in the future should a suitable partner for this undertaking be identified. Their update in June 2023 stated that 8/14 participants expressed improved vision-related quality of life (QoL) (Ref 89). Recently, the group published the latest status of the BRILLIANCE phase 1–2 study in early May 2024 in the New England Journal of Medicine (Ref 90). According to the report, 12 adults (17–36 years of age) and 2 children (9 and 14 years of age) were injected with varying doses (low, intermediate, high) of EDIT-101. No serious adverse effects related to the treatment or procedure, or dose-limiting toxic reactions were reported. Briefly, 6 participants displayed a meaningful improvement from baseline in cone-mediated vision; meaningful progress from baseline in the best-corrected visual acuity was reported in 9 participants; and improvement from baseline in the vision-related QoL score was evident in 6 participants.

Thus CRISPR holds promise for potentially treating genetic blindness by targeting and correcting mutations associated with the condition. While initial safety data for EDIT-101 may be promising, uncertainties persist regarding its long-term safety. Delivery of this treatment via a viral vector implies sustained expression of CRISPR-Cas components within the eye, thereby increasing the risk of unintended DNA alterations and potential immune reactions to the viral vector or the Cas protein over an extended period. Monitoring these patient volunteers over several years will be imperative to assess their long-term outcomes, as the potential for unintended genetic changes, such as off-target effects or genomic instability, underscores the need for extended monitoring to assess risks that may not manifest immediately but could have significant consequences over time.

Currently, there is no direct method available to evaluate the percentage of edited cells or identify unintended edits. Evaluation of editing efficiency can only be inferred based on observed improvements in vision among patient volunteers. Researchers actively monitor individuals who have received treatment to determine the stability, progression, or regression of vision improvements over time. Variability in editing outcomes could impact the overall efficacy of the treatment, particularly in a condition like LCA10, where the restoration of function in retinal cells requires high accuracy and uniformity. Furthermore, the researchers acknowledge that the results of their study support the safety of the treatment to the extent that it can be assessed in a small number of patients, as it sets limitations to the interpretation of the data and presents challenges for drawing robust conclusions (Ref 90). Addressing these critical aspects will allow future studies to build a more comprehensive understanding of the risks and benefits associated with genome editing in clinical applications for LCA10.

### Genetic modification of stem cell-derived pancreatic cells for diabetes

Type 1 diabetes (T1D) is characterised by autoimmune destruction of pancreatic beta cells and consequent inadequate levels of insulin secretion. Vigilant management of blood sugar and insulin levels throughout a lifetime is necessary. Common serious complications of T1D encompass kidney damage, nerve pain, vascular and cardiac issues, vision impairment and limb amputation. Pancreatic islet transplantation has proven effective in treating individuals with unstable, high-risk T1D. However, this procedure is associated with a scarce supply of donor organs and the complexities of obtaining consistent and reliable islet preparations. Although ongoing clinical trials indicate substantial benefits from pancreatic cell transplantation, recipients of conventional transplants necessitate continual immune system suppression to avert rejection. The use of immunosuppressant drugs poses serious risks, including an elevated susceptibility to infections and cancers. A successful replacement therapy using stem cell-derived islets has the potential to overcome these challenges, offering a solution that could serve a larger number of people to constitute an effective alternative to the other treatment methods (Ref 91).

**Figure 7.**
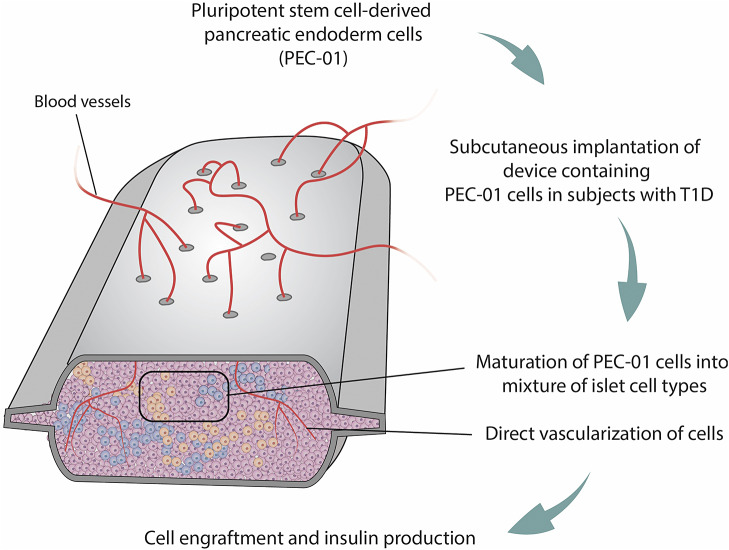
Schematic representation of VC-02 Macroencapsulation Device (Ref 91). The VC-02 macroencapsulation device is designed to encapsulate and protect insulin-producing cells for transplantation into individuals with type 1 diabetes (T1D). The encapsulation provided by the VC-02 device helps to maintain the viability and function of the transplanted cells. This can lead to more stable and consistent insulin production, which aids in better controlling blood sugar levels in individuals with T1D. By providing immune protection, the VC-02 device may reduce or eliminate the need for immunosuppressive drugs, typically required to prevent rejection in traditional islet cell transplantation.

Results from a phase 1/2 open-label trial, the first of its kind conducted in humans, offer compelling evidence that pluripotent stem cell-derived pancreatic endoderm cells (PEC-01) transplanted into individuals diagnosed with T1D transform into islet cells capable of releasing insulin and c-peptide in a manner that mimics natural physiological regulation (Ref 91). Study participants were administered immunosuppressive medications to support the growth of these cells and prevent rejection by the body’s immune system of the implanted VC-02™ macro-encapsulation devices (Figure 7). This system holds refinements for increased engraftment and insulin production via direct vascularisation by the host vasculature, compared to the earlier VC-01 immuno-isolating units which depended on semipermeable membranes that were cell impermeant (Ref 92). In this research, which involved 17 subjects aged between 22 and 57, all diagnosed with T1D, PEC-01 cells were subcutaneously implanted into VC-02 units facilitating direct vascularisation. Early clinical results reveal that following the implantation and successful engraftment, the PEC-01 pancreatic progenitor cells undergo maturation into human endocrine islet tissue. Throughout the clinical trials conducted thus far, ViaCyte’s product candidates have exhibited strong tolerability with minimal side effects related to the product. Both histological evidence and measurements of c-peptide (insulin) production confirm the intended functionality of PEC-01 cells following engraftment.

Taking this strategy further, in February 2022, CRISPR Therapeutics and ViaCyte performed the first-in-human transplant of the CRISPR-edited, stem cell-derived pancreatic cells for T1D treatment (CTX210A). In this innovative approach, CRISPR technology is utilised to modify immune-related genes within pancreatic cells derived from pluripotent stem cells, rendering them impervious to the patient’s immune system (Ref 93). The ultimate goal is to furnish patients with robust, new pancreatic cells capable of managing or potentially curing T1D without the need for chronic immunosuppression. CRISPR Therapeutics and ViaCyte sponsored this phase 1 trial, marking the initial application of CRISPR for treating an endocrine disease. Shortly after the initial dosing of the first patient in spring 2022, Vertex Pharmaceuticals acquired ViaCyte. Yet on 8 January 2024, Vertex announced it would cut ties to the T1D stem cell therapy by CRISPR Therapeutics. It appears that CRISPR Therapeutics is now planning to hold a phase I/II trial of the now-called CTX211 as the next-generation drug candidate, which they define as an ‘investigational allogeneic, gene-edited, immune-evasive, stem cell-derived beta-cell replacement therapy’ in their pipeline.

This method could provide patients with the advantages of transplantation, even potentially curing T1D, without encountering the risks and side effects linked to immunosuppressive drugs. CRISPR/Cas9 technology is often utilised in the hypoimmunogenic induced pluripotent stem cell (iPSC) line development, as a future potential universal source of ‘off-the-shelf’ cells to be used in allogeneic cell therapy (Ref 94). Although providing easy and successful modification of the target cells, the requirement of several alterations for the process brings along a high probability of off-target effects, which remain to be addressed thoroughly in clinical studies (Ref 95). Overall, the pivotal outcome of these trials will be whether the edited cells can effectively evade detection by the immune system, a critical factor in determining the success of the treatment.

### Gene editing tools against HIV

Human Immunodeficiency Virus (HIV) is a viral infection that attacks the immune system by infecting CD4 (helper) T lymphocytes. Reproducing within the CD4 cells, HIV leads to cell death and the release of more viruses to infect and eliminate other helper T cells. If left untreated, HIV can progress to acquired immunodeficiency syndrome (AIDS), causing severe immune system damage and leaving individuals susceptible to common infections that can lead to serious illness or death. Those with AIDS also face increased vulnerability to rare infections and cancers uncommon in individuals with healthy immune systems.

Excision Biotherapeutic’s EBT-101 is a unique experimental *in vivo* CRISPR-based treatment developed to provide a one-time intravenous infusion for the treatment of HIV infections (Ref 96). Using an adeno-associated virus-9 (AAV9), EBT-101 transports CRISPR-Cas9 and dual guide RNAs, employing a multiplex editing technique that targets three specific locations within the HIV genome. This enables the removal of significant segments of the HIV genome, reducing the likelihood of viral escape. This is the first instance of a CRISPR-based therapy administered for infectious disease, as well as being the first to target a retrovirus. Researchers employed COTANA (CRISPR-Off-Target Nomination and Analysis) to steer CRISPR-Cas9 editing, creating sets of gRNAs that precisely target HIV without bearing significant resemblance to locations in the human genome. A subsequent analysis using multiplex amplicon sequencing demonstrated the effective removal of a substantial portion of the viral genome without any unintentional insertions or deletions in the genomic DNA.

Sponsored by Excision Biotherapeutics, this trial was granted Fast Track Designation by the FDA in July 2023. As a phase 1/2 trial, its objectives included assessing safety and side effects, determining the correct dosage, and evaluating the treatment’s efficacy in excising the virus from CD4 cells in individuals living with HIV Type I, constituting nearly 95% of the prevalence worldwide (Ref 97). The first participant was dosed in September 2022. In October 2023, at the European Society of Gene and Cell Therapy Congress (ESGCT), the company presented favourable safety and biodistribution findings for up to 48 weeks, based on the results from three patients dosed safely, experiencing no adverse events or dose-limiting toxic effects.

Before the clinical trial, EBT-101 displayed curative potential in mice and macaque monkeys, as announced by Excision Biotherapeutics in a press release. However, it was a concern that the percentage of latently infected cells in humans would be much lower than that modelled in cell cultures and animal subjects (up to 100%), as a factor to reduce the efficacy of the eradication process (Ref 98). The clinical data from this trial, presented recently at the American Society of Gene and Cell Therapy (ASGCT) meeting in May 2024, in fact, revealed that HIV viral suppression was not maintained at the initial dose tested, possibly because EBT-101 failed to reach all the cells with latent HIV in the 5 patients dosed. What is planned next throughout the course is yet to be announced (Ref 99).

In a novel approach to combat HIV infection, mature primary B cells from mice and humans were edited *in vitro* using CRISPR/Cas9 to express mature neutralising antibodies (bNAbs) from the endogenous immunoglobulin heavy chain (Igh) locus (Ref 100). The modified B cells retained their capacity to take part in humoral immune responses. Wild-type mice that received these edited B cells and were immunised with the corresponding antigen exhibited HIV-1-neutralising bNAb titers sufficient to protect against infection, facilitating humoral immune responses that might be challenging to achieve through conventional immunisation methods. This is a promising application where advanced gene editing is combined with immunology, creating a cutting-edge strategy in the fight against HIV infection.

Although effective gene editing approaches raise hope in the combat against HIV, challenges remain, such as HIV-1’s high mutation rate, besides the common issues such as the off-target effects, immunogenicity, and delivery of the large CRISPR/Cas9 complex. The high specificity required for safely targeting HIV without compromising host cell integrity remains a significant technical barrier. The need to effectively target a sufficient number of cells to eliminate the disease makes this task more complex than treating conditions such as blood disorders. This incomplete targeting can allow residual viral reservoirs to persist, potentially leading to viral rebound if treatment is halted. Furthermore, the *in vivo* CRISPR editing strategies against HIV infection lead to the prolonged presence and widespread distribution of genome-editing components, heightening the risk of unwanted edits and immune reactions. Participants in related trials will undergo long-term monitoring to assess any potential health effects associated with unintended DNA alterations (Refs 101, 102). Refining the specificity of guide RNAs and minimising off-target activity through advanced editing technologies will be crucial for translating this approach into a safe and effective therapy for HIV.

### Lipid nanoparticle-mediated targeted delivery of genome editing tools against protein folding disease

**Figure 8.**
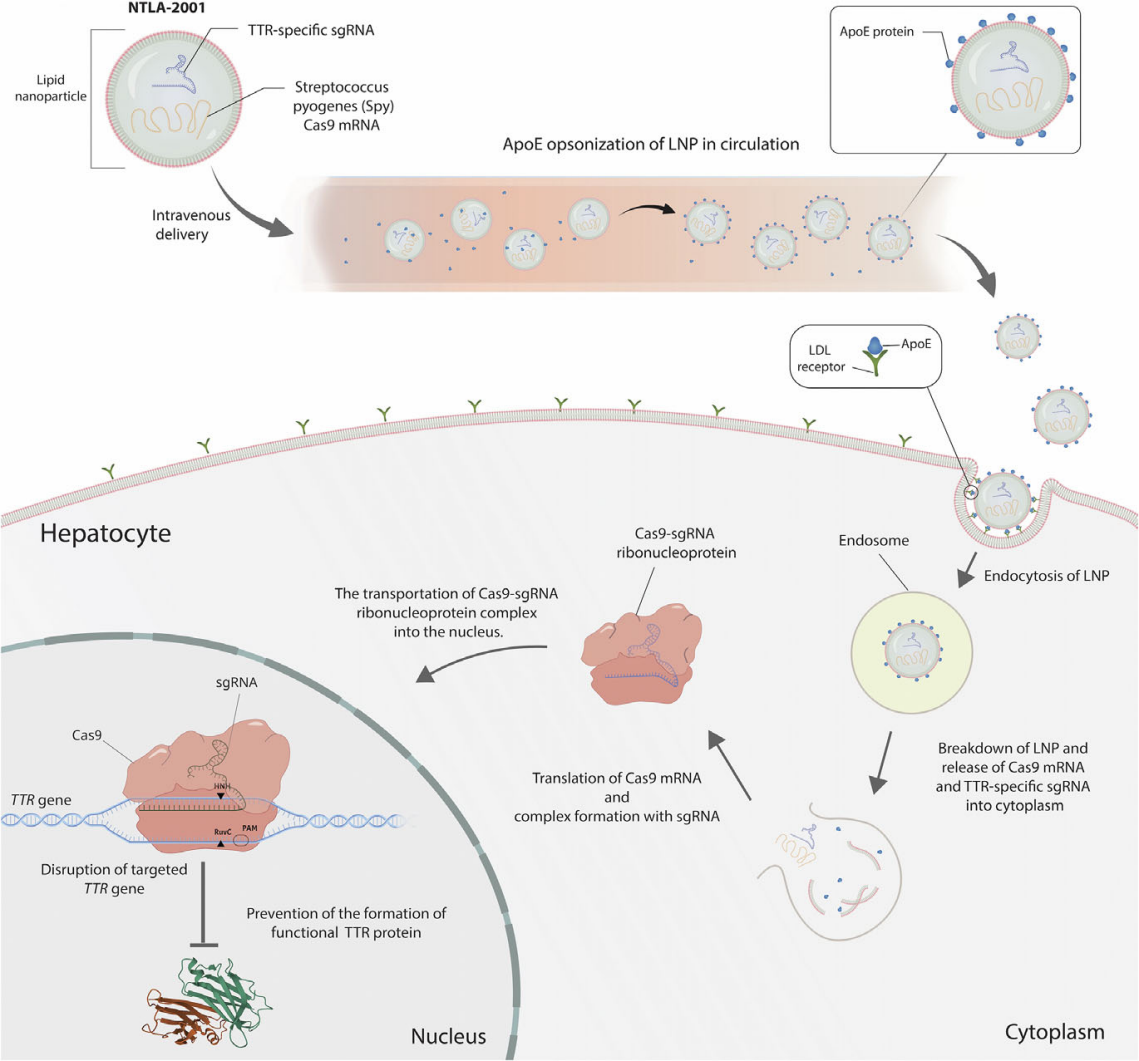
The mechanism of *in vivo* gene editing for Transthyretin Amyloidosis (Ref 106). NTLA-2001 employs a lipid nanoparticle (LNP) as its carrier system. The active ingredients of NTLA-2001 consist of a human-optimised messenger RNA (mRNA) molecule encoding the *Streptococcus pyogenes* (Spy) Cas9 protein and a single guide RNA (sgRNA) molecule targeting the human gene responsible for transthyretin (TTR) production. After NTLA-2001 is administered intravenously and enters the bloodstream, the LNP becomes opsonised by apolipoprotein E (ApoE) and is then transported through the systemic circulation directly to the liver. The NTLA-2001 lipid nanoparticle (LNP) is absorbed by hepatocytes via the surface LDL receptors and undergoes endocytosis. Subsequent to the breakdown of the LNP and the disruption of the endosomal membrane, the active constituents, namely the TTR-specific single guide RNA (sgRNA) and the messenger RNA (mRNA) encoding Cas9, are liberated into the cytoplasm. The Cas9 mRNA is then translated via the standard ribosomal process, leading to the generation of the Cas9 endonuclease enzyme. The TTR-specific sgRNA engages with the Cas9 endonuclease, thereby forming a CRISPR–Cas9 ribonucleoprotein complex. The Cas9 ribonucleoprotein complex is targeted for nuclear import, and it subsequently enters the nucleus. The 20-nucleotide sequence at the 5′ end of the sgRNA binds to the target DNA, enabling the CRISPR-Cas9 complex to access the gene and induce precise DNA cleavage at the TTR sequence through conformational changes and nuclease domain activation. Endogenous DNA repair mechanisms then join the cut ends, potentially causing insertions or deletions of bases (indels). The formation of an indel may lead to reduced levels of functional mRNA for the target gene due to missense or nonsense mutations, ultimately resulting in decreased production of the target protein.

Transthyretin (TTR) is a transport protein found in both plasma and cerebrospinal fluid, dedicated to transporting the thyroid hormone thyroxine (T4) and retinol to the liver. TTR is released by the liver into the bloodstream and to the cerebrospinal fluid by the choroid plexus. Transthyretin amyloidosis, also known as ATTR amyloidosis, is an uncommon, progressive and fatal disease. Hereditary ATTR amyloidosis (ATTRv amyloidosis) arises when mutations in the *TTR* gene are present from birth, causing the liver to produce structurally abnormal TTR proteins tending to misfold (Ref 103). These faulty proteins accumulate as amyloid deposits throughout the body, resulting in severe complications affecting various tissues such as the heart, nerves, and the digestive system. ATTRv amyloidosis commonly presents as polyneuropathy (ATTRv-PN) causing nerve damage or cardiomyopathy (ATTRv-CM) leading to heart failure. NTLA-2001 is an *in vivo* gene-editing tool targeting ATTR amyloidosis leveraging the CRISPR/Cas9 technology to reduce serum TTR concentrations (Figure 8) (Ref 104). It is the first investigative CRISPR therapy candidate designed for systemic administration, delivered intravenously as a single-dose treatment to execute gene editing within the human body. Intellia’s exclusive non-viral platform employs lipid nanoparticles (LNPs) for the targeted delivery of a two-part genome editing system to the liver. This system includes customised gRNA designed for the disease-associated gene and messenger RNA encoding the Cas9 enzyme responsible for precise editing. Extensive preclinical data display a significant and enduring reduction in TTR levels following *in vivo* inactivation of the target gene (Ref 105).

Conducted by Intellia and spanning sites in the EU, UK and New Zealand, the trial initiated dosing its first participants in late 2020 and is bifurcated into two arms (Ref 106). One arm focuses on patients presenting neuropathy symptoms, while the other targets those with symptoms of cardiomyopathy. Across both arms, data has been collected from 27 participants receiving varying doses. Remarkably, even at the lowest treatment dosage, a substantial reduction (>85%) was reported in toxic protein levels in participants’ bloodstreams, with those at the highest dose experiencing a reduction exceeding 90%. Sustained reduction in TTR protein has been observed over time for all patients, including those for whom a year of findings has been disclosed. Given the correlation between TTR protein levels and disease severity, researchers hold optimistic expectations for participant outcomes. Although some infusion-related side effects were observed, they were temporary and of a non-severe nature (Refs 53, 106). The treatment’s FDA clearance to start a pivotal Phase 3 trial of NTLA-2001 came in October 2023. In November 2023, the company shared updated data from over 60 patients included in the Phase I study; deep and durable serum TTR reduction was evident via a single dose of NTLA-2001, including the initial 29 patients, followed up for 12 months or longer. The drug was generally well tolerated across both arms of trials. The company announced a redosing in June 2024 with a press release, stating a 90% median reduction in serum TTR levels at day 28 in three patients who received the lowest dose in the previous Phase 1 dose-escalation. The company specifies that the MAGNITUDE trial (NCT06128629), which is currently recruiting, will be conducted as a randomised, double-blind, placebo-controlled study to evaluate the safety and efficacy of the drug in 765 patients.

Although an effective strategy, additional research on the long-term safety and effectiveness of NTLA-2001, especially in higher-risk patients, is crucial. This includes continued monitoring to determine if knocking out the *TTR* gene with this approach leads to a sustained reduction of TTR levels over an extended period. Assessing the suitability of this technology for other eligible diseases will also be significant (Ref 107).

### Gene disruption technology to stop inflammatory disease

In hereditary angioedema (HAE), individuals experience severe episodes of inflammation resulting in swelling, typically affecting the arms and legs, face, intestines, or airway. While intestinal swelling may lead to intense pain, nausea, and vomiting, swelling in the airway may present a life-threatening risk (Ref 108). HAE attacks typically begin during childhood, and if left untreated, tend to reoccur every 1–2 weeks, each episode lasting for 3–4 days. It is a rare disease affecting approximately 1 in every 50,000 to 1 in every 100,000 individuals. Three distinct categories of HAE are acknowledged, and Types I and II are linked to genetic mutations that affect the production of the C1 inhibitor protein (C1-INH), a serine protease inhibitor that plays a critical role in regulating the kallikrein-kinin system (Ref 109). Type I HAE is caused by mutations in the *SERPING1* gene, leading to reduced levels of functional C1-INH protein. Type II HAE is also caused by *SERPING1* mutations but results in normal or elevated levels of a dysfunctional C1-INH protein. HAE with normal C1 inhibitor (HAE-nC1-INH) is a form of HAE where the levels and function of C1-INH are normal. Unlike Type I and Type II HAE, which are caused by mutations in the *SERPING1* gene affecting C1-INH, HAE-nC1-INH is associated with mutations in other genes that disrupt the regulation of bradykinin or related pathways. This includes subtypes associated with mutations in genes such as *FXII*, *PLG*, or *ANGPT1*, or cases without identified genetic mutations (Ref 110).

In individuals with a healthy immune system, precise coordination of proteins regulates inflammation, enabling the body to react effectively to threats and injuries. The C1 inhibitor protein plays a pivotal role in suppressing inflammation. However, when C1 inhibitor protein levels are reduced, as in HAE, the bradykinin protein accumulates in the bloodstream. Excess bradykinin, in turn, causes fluid to escape from blood vessels into the body tissues, initiating HAE swelling attacks. Current treatment options include daily oral medications or administration via IV infusions or injections, sometimes needed as frequently as twice a week. Despite regular administration, individuals with HAE may still encounter occasional attacks. Similar to hATTR, angioedema can be acquired but also may be inherited (Ref 111).

NTLA-2002 is a CRISPR drug candidate developed by Intellia Therapeutics for HAE, intended to target the *KLKB1* gene in liver cells to reduce kallikrein protein production (Ref 112). The excessive activity of kallikrein results in the overproduction of bradykinin, causing recurrent, severe and potentially life-threatening swelling attacks in HAE. Reduced bradykinin levels provided via lowered kallikrein activity correlate with decreased inflammation and swelling. Administered through a single IV dose, the objective is gene disruption to halt the progression of the disease. Throughout the process, DSB damage is generated in the *KLKB1* target gene, and further mutations are initiated as the cell attempts to repair the damage without a corrected template. Severe damage in the gene ultimately may lead to cessation of protein production. In this trial, CRISPR-Cas9 reagents are delivered via LNPs to edit cells in the liver, leveraging the natural tendency of LNPs to accumulate in the liver, thus ensuring precise targeting. The NTLA-2002 therapy shows promise for Type I and II HAE but has limited applicability for HAE with normal C1-INH (e.g., HAE-FXII), as the applicability of this drug to nC1-INH HAE depends on whether kallikrein overproduction plays a significant role in the pathophysiology. Some patients with nC1-INH may not benefit if their swelling episodes are not driven by the kallikrein-bradykinin pathway.

In New Zealand, a range of three doses was administered to 10 participants, and the extended follow-up data has reached over 2 years in the earliest patients dosed. According to Intellia Therapeutics’ update on 2 June 2024, the majority of the patients remained attack-free for over 18 months or longer, with the longest attack-free interval reported as over 26 months for an individual patient post-application. Plasma kallikrein reduction was 60% for the low dose (25 mg), 88% for the medium dose (50 mg), and 95% for the high dose (75 mg) NTLA-2002 application. The treatment has shown good tolerance across all dosage levels, with no severe adverse events (Ref 113). Intellia has recently (22 January 2025) announced the dosing of the first subject in their Phase III trial of NTLA-2002. Termed ‘HAELO’, this randomised, double-blind, placebo-controlled study aims to determine the safety and efficacy of the drug in 60 adults with Type 1 or Type II HAE. The five regulatory designations received by the drug at this time are listed as Orphan Drug (September 2022) and RMAT (March 2023) Designations by the FDA, the Innovation Passport by the UK Medicines and Healthcare products Regulatory Agency (MHRA) (January 2023), Priority Medicines (PRIME) Designation by the European Medicines Agency (October 2023), and Orphan Drug Designation by the European Commission (November 2023) (Ref 114).

While early results from these trials are encouraging, several challenges remain, including the conclusions yet to be driven from a small number of patients. The recently initiated Phase III trial, involving 60 patients, will provide safer conclusions to be drawn in this regard. Other risks include potential off-target effects and immune response to delivery methods like LNPs, which could impact efficacy or cause inflammation. The long-term safety of sustained kallikrein reduction is also unclear. Manufacturing scalability is another concern due to the complexity of mass-producing CRISPR components like Cas9 and guide RNA, coupled with high costs that may limit accessibility. Delivery poses challenges in ensuring precise targeting to the liver and addressing variability in patient factors such as liver health and genetics. Further optimisation is needed to improve safety, delivery, affordability, and broader applicability (Ref 115).

### Bacteriophage therapy involving CRISPR-Cas3 for chronic infection

Urinary tract infections (UTIs) are prevalent complications leading to more than 8 million healthcare provider visits annually. The primary culprit is typically *E. coli*, a common faecal bacterium. UTIs often present with symptoms such as a burning sensation during urination and frequent drives to urinate (Ref 116). In addition to causing discomfort, these infections can become a concern if they progress to affect the kidneys or if bacteria manage to enter the bloodstream. While most UTIs respond well to a brief antibiotic course, there are instances where antibiotics prove ineffective or the infection persists, referred to as chronic UTIs (Ref 117).

Bacteriophages, commonly called phages, are viruses that attack, infect and replicate in bacteria. Their typical mode of action involves injecting genetic material into bacteria and utilising them as factories to generate more phages. Ultimately, the bacteria may undergo bursting, releasing additional copies of the phage. Phages are currently being explored for their potential use against bacterial infections, gaining increased attention in response to the escalating threat of antibiotic resistance. Although the concept dates back about a century, the advent of antibiotics like penicillin and challenges in patenting phages impeded its therapeutic development.

Over the past few decades, phages have been utilised in ‘compassionate treatment’, which involves the use of an unapproved drug or therapy to treat severely ill individuals when no other treatment options are available (Ref 118). Differing degrees of success were reported in around 25 documented instances in the last 20 years, although clinical trials are required to evaluate safety and efficacy (Ref 118). Phages may offer a distinct advantage of targeting specific types of bacteria, while antibiotics can harm healthy bacteria without discrimination. Thus, phage therapy has the potential for more specific and accurate interventions.

The clinical trial ELIMINATE conducted by Kim et al. utilises CRISPR technology to develop phage therapy against uncomplicated UTIs, as the first rigorously controlled trial in the field (NCT05488340) (Ref 119). In this innovative strategy involving the CRISPR-enhanced six-bacteriophage cocktail drug LBP-EC01, bacteriophages are modified to boost their effectiveness against *E. coli* via a CRISPR-Cas3 system incorporated into their genome for DNA-targeting activity. Experimental findings from animal models with urinary tract and other infections demonstrate that CRISPR-mediated modifications significantly enhance the phages’ ability to eliminate *E. coli* (Ref 120). LBP-EC01 is carefully designed to target the genomes of three *E. coli* strains responsible for greater than 80% of UTIs, regardless of the antibiotic drug resistance status of the bacteria (Refs 119, 121).

During the phase 1 trial, Locus Biosciences administered the treatment directly to the bladder through a catheter. In February 2021, a Phase 1b trial was completed in the United States, confirming the innovative therapy’s safety and tolerability without any drug-related adverse effects (Ref 122). In 2022, Locus initiated the enrolment of participants for a phase 2/3 trial to test the preliminary efficacy of the drug, with the dosing of the first participant officially announced in September 2022. The aim is to recruit around 800 participants from the United States and the European Union (Ref 123). An update published by the researchers in the Lancet Infectious Diseases in December 2024 reported outcomes from the Part 1 dose regimen selection portion of a 2-part trial examining LBP-EC01, from 39 patients between the ages of 18 and 70, enrolled between August 2022 and August 2023 (Refs 121, 124). The trial was held in 6 private clinical sites in the USA. A treatment regimen involving 2 days of intraurethral LBP-ECO1 and 3 days of concurrent LBP-ECO1 intravenous administration along with the oral application of trimethoprim-sulfamethoxazole (TMP-SMX) twice a day was reported to be well-tolerated. Consistent pharmacokinetic profiles in blood and urine were specified in the report, with the treatment providing a fast and durable reduction of *E. coli* and the clinical symptoms eliminated in evaluable patients.

Although phage therapy is considered safe so far, evaluating possible side effects of phage accumulation will need more studies. Bacteria may possess various mechanisms for evading killing by the phages, though it is argued that bacterial mechanisms used for evasion of killing by bacteriophages may also have an overall reducing effect on their virulence and fitness in the patient. This may be a means of making these treatments successful even in the presence of resistance and even ‘steering’ these bacteria back to states of antibiotic susceptibility (Refs 125, 126).

### Editing genes for cardiovascular disease through genetic interruption

Increased low-density lipoprotein cholesterol (LDL-C) has been strongly associated with cardiovascular diseases (CVDs) by many epidemiological and interventional studies as a major risk factor (Ref 127). The influence of genetics on cholesterol levels is evident in individuals with mutations in the proprotein convertase subtilisin/kexin type 9 (*PCSK9*) gene, leading to familial hypercholesterolaemia (FH). FH is a hereditary condition characterised by dangerously high cholesterol levels irrespective of diet and exercise. As a result, plaque accumulates in the arteries, leading to reduced blood flow or blockage (Ref 128). In 2022, Verve Therapeutics initiated a trial targeting patients who are heterozygous for a high-risk subtype of FH (HeFH) with established atherosclerotic cardiovascular disease (ASCVD) and uncontrolled levels of LDL-C, using a lipid nanoparticle (LNP)-delivered base editor system (NCT05398029) (Ref 129). In this specific trial, the *in vivo* liver base editing medicine (VERVE-101) is designed to introduce a single-letter change in the *PCSK9* gene to turn off the disease-causing gene permanently (Ref 130).

The initial participant in the phase 1b clinical trial Heart-1 received the treatment in July 2022, only 6 years after Harvard University researchers invented base editing (Refs 25, 129). Two more participants were dosed by October 2022, and no serious side effects have been noted (Ref 131). Meanwhile, the FDA has placed a clinical hold on the Investigational New Drug (IND) application for VERVE-101, thereby delaying the initiation of a clinical trial for this therapeutic. The FDA’s directive was rooted in the need for Verve to furnish additional preclinical data concerning potency differences between human and non-human cells, potential risks associated with editing germline cells, as well as off-target studies in non-hepatocyte cell types, and available clinical data from the ongoing trial. Having met the requirements, the FDA lifted the clinical hold on VERVE-101 in October 2023 (Ref 132).

The company reported significant trial findings, indicating a time-averaged reduction in blood PCSK9 levels ranging from 39% to 84% across different doses. Patients in the two higher-dose groups experienced treatment-related adverse effects, including infusion reactions (which were transient and ranged from mild to moderate), temporary asymptomatic increases in liver transaminases, below the upper normal limits of bilirubin, and serious cardiovascular events in those with severe underlying ASCVD. Thirteen patients were dosed in total, with the 3 additional patients dosed in April 2024. In 2 patients with the longest follow-up in the higher-dose cohorts, LDL-C reduction was maintained for 270 days, with the follow-up ongoing. However, Verve has decided to pause enrolment in the trial due to VERVE-101-associated laboratory abnormalities to conduct an investigation (Ref 133). The Clinical Trial Applications (CTAs) in the UK and New Zealand and the Investigational New Drug Application (IND) in the US were announced to be active.

Verve Therapeutics also reports a second *PCSK9* gene editor, VERVE-102, developed similarly for *PCSK9* inactivation like VERVE-101, but to be delivered using their proprietary GalNAc-LNP technology. This system allows access to the LNPs to deliver the drug to liver cells via either the asialoglycoprotein receptor (ASGPR) or the low-density lipoprotein receptor (LDLR). It comprises an adenine base editor-expressing messenger RNA and an optimised RNA targeting *PCSK9.* The drug is currently tested in the Heart-2 open-label Phase 1b clinical trial in two patient populations: adults with heterozygous familial hypercholesterolaemia (HeFH) and adults with premature coronary artery disease (CAD). In May 2024, the company announced the dosing of the first patient in the Heart-2 Phase 1b clinical trial where VERVE-102 is being evaluated. As of October 2024, the company already reported 7 participants dosed in the Heart-2 clinical trial across two cohorts.

Verve Therapeutics’ VERVE-201, on the other hand, is an investigational CRISPR base editing tool targeting the inactivation of the *ANGPTL3* gene in liver cells via alteration of a single DNA base, thus turning off its production by the liver to reduce LDL-C and triglyceride levels. Preclinical data reveals on-target precise and potent editing in primary human hepatocytes, *Ldlr^−/−^* and wild-type mice, and non-human primates, displaying its potential in treating severe or complete LDLR deficiency that is evident in homozygous FH (HoFH). The first participant in their clinical trial for VERVE-201 was recently announced to be dosed in November 2024 (Ref 134). The challenge with LNP-mediated delivery to the liver is a major problem for patients with HoFH due to complete deficiency in the LDLR in these patients. This problem is overcome by the use of GalNAc-lipid nanoparticles to enable non-LDLR-dependent hepatic delivery (Refs 135, 136).

## An overall look, challenges, and future prospects

Clinical trials of CRISPR-mediated gene editing represent a groundbreaking frontier in biomedical research, offering unprecedented potential for targeted treatments of genetic disorders and diseases. Various trials utilising CRISPR for gene editing in therapeutic contexts have yielded promising results, culminating in the recent announcement of Casgevy as the first FDA-approved CRISPR-based medication (Ref 54). With extensive research in the field and ongoing clinical trials, many new drugs are expected to receive approval in the upcoming decades (Ref 137). Overall, gene editing-mediated clinical trials showcase diverse applications (Table 1). Of these, the most prominent CRISPR-mediated trials are discussed in the text in relevant sections. In this section we take an overall look with an emphasis on current challenges and possible solutions, recent advancements, and future prospects.

**Table 1.** **Gene editing-based clinical trials** (Refs 138, 139). Information was gathered from clinicaltrials.gov accessed on 10 January 2025. The ‘NCT Number’ column provides the unique identifier assigned to the clinical trial on clinicaltrials.gov. HbF: Foetal Haemoglobin; hHSPCs: Human Haematopoietic Stem and Progenitor Cells; SCD: Sickle Cell Disease; TBT: Transfusion-Dependent β-Thalassaemia: tBE: Transformer Base Editing; HAE: Hereditary Angioedema

Disease area	Condition	Trial ID	Treatment details	Sponsor	Phase	Status
Autoimmune diseases	Kabuki Syndrome 1	NCT03855631	CRISPR-Cas9: *Ex vivo* therapy targeting KMT2D via correction to address developmental anomalies in Kabuki syndrome.	Montpellier Hospital	Not Applicable	Completed
	Type 1 Diabetes	NCT05210530	CRISPR-Cas9: *Ex vivo* therapy targeting pancreatic beta cells via gene correction to restore insulin production.	CRISPR Therapeutics AG	Phase 1	Completed
		NCT05565248	VCTX211: *Ex vivo* CRISPR-Cas9-modified PEC211 pancreatic endoderm cells for immune evasion, delivered via a perforated cell-retention device.	CRISPR Therapeutics AG	Phase 1/2	Recruiting
Cardiovascular diseases	Hypercholesterolaemia	NCT05398029	VERVE–101, a base-editing therapy targeting the PCSK9 gene in the liver to reduce LDL-C and PCSK9 levels. Open-label, single-ascending dose Phase 1b study.	Verve Therapeutics, Inc.	Phase 1	Active
		NCT06164730	VERVE–102: *In vivo* adenine base editing therapy targeting PCSK9 via gene knockout to reduce LDL cholesterol levels.	Verve Therapeutics	Phase 1	Recruiting
		NCT06451770	VERVE–201: *In vivo* adenine base editing therapy targeting ANGPTL3 to inactivate gene expression and lower circulating LDL cholesterol (LDL-C).	Verve Therapeutics	Phase 1	Recruiting
Genetic disorders	Alpha–1 Antitrypsin Deficiency	NCT06622668	NTLA–3001: *In vivo* CRISPR-Cas9 therapy targeting SERPINA1 to knock out mutant alpha–1 antitrypsin protein production, aiming to prevent lung damage.	Intellia Therapeutics	Phase 1	Recruiting
	Eye Diseases	NCT03872479	EDIT–101: *In vivo* therapy targeting CEP290 via correction to restore photoreceptor function.	Editas Medicine Inc.	Phase 1/2	Ongoing
		NCT06031727	HG202: CRISPR/Cas13 RNA-editing therapy for treating Neovascular Age-related Macular Degeneration (nAMD) by knocking down VEGFA expression to inhibit CNV formation.	HuidaGene Therapeutics Co., Ltd.	Phase 1	Recruiting
		NCT05805007	EDIT–103: *In vivo* CRISPR-Cas9 therapy targeting RHO gene mutations to prevent retinal degeneration and preserve vision in patients with Retinitis Pigmentosa.	Editas Medicine	Early Phase 1	Recruiting
	Chronic Granulomatous Disease (CGD)	NCT06559176	PM359: Prime Editing gene therapy for CGD caused by NCF1 gene mutations. Autologous CD34+ cells are edited *ex vivo* to correct the delGT mutation.	Prime Medicine, Inc.	Phase 1/2	Recruiting
Haematological disorders	Severe Sickle Cell Disease	NCT04774536	CRISPR-SCD001: *Ex vivo* CRISPR-Cas9 therapy targeting haematopoietic stem cells to reactivate HbF and alleviate symptoms of sickle cell disease.	Mark Walters, MD	Phase 1/2	Recruiting
		NCT05456880	BEAM–101: Autologous CD34+ HSPCs edited via base editing to increase HbF.	Beam Therapeutics Inc.	Phase 1/2	Recruiting
		NCT05329649	CTX001: *Ex vivo* CRISPR-Cas9 therapy using autologous modified CD34+ hHSPCs to evaluate safety and efficacy in paediatric participants with severe SCD.	Vertex Pharmaceuticals	Phase 3	Recruiting
		NCT03745287	CTX001: *Ex vivo* CRISPR-Cas9 therapy using autologous CD34+ hHSPCs modified at the erythroid lineage-specific enhancer of the BCL11A gene to evaluate safety and efficacy in subjects with SCD.	Vertex Pharmaceuticals	Phase 2/3	Active
		NCT05951205	Exa-cel (CTX001): *Ex vivo* CRISPR-Cas9 therapy using autologous CD34+ hHSPCs in participants with severe SCD, βS/βC Genotype.	Vertex Pharmaceuticals	Phase 3	Ongoing
		NCT06287099	BRL–101: *Ex vivo* CRISPR-Cas9 therapy using autologous CD34+ hHSPCs to evaluate safety and efficacy in SCD.	BioRay Laboratories	Not Applicable	Ongoing
		NCT03653247	BIVV003: Autologous HSC transplantation using gene-edited cells; Plerixafor and Busulfan used for conditioning.	Sangamo Therapeutics	Phase 1/2	Active
		NCT04443907	OTQ923: *Ex vivo* CRISPR-Cas9 therapy targeting BCL11A in HSPCs to increase HbF and reduce sickling complications.	Novartis Pharmaceuticals	Phase 1	Active
		NCT06565026	CS–206: Autologous CD34+ cells edited via *in vitro* base editing targeting BCL11A to restore HbF.	CorrectSequence Therapeutics Co., Ltd	Phase 1	Recruiting
	Sickle Cell Disease and Beta Thalassaemia	NCT05477563	CTX001: *Ex vivo* CRISPR-Cas9 therapy using autologous CD34+ hHSPCs to evaluate safety and efficacy in participants with TBT and SCD	Vertex Pharmaceuticals	Phase 3	Recruiting
	Beta-Thalassaemia	NCT03432364	ST–400: Autologous haematopoietic stem cells edited with ZFN to disrupt the BCL11A enhancer, boosting HbF. Patients undergo conditioning chemotherapy before infusion.	Sangamo Therapeutics	Phase 1/2	Completed
		NCT03728322	Gene-corrected patient-specific induced haematopoietic stem cells (iHSCs) using CRISPR/Cas9 to correct HBB mutations. The safety and efficacy of transplantation are being investigated.	Allife Medical Science and Technology Co., Ltd.	Phase 1	Unknown
		NCT05444894	EDIT–301: *Ex vivo* CRISPR-Cas12a therapy using autologous CD34+ HSPCs to evaluate safety, tolerability, and efficacy.	Editas Medicine	Phase 1/2	Recruiting
		NCT06041620	VGB-Ex01: *Ex vivo* CRISPR-Cas12b therapy editing the HBG1/2 promoter to reactivate gamma-globin, induce HbF, and reduce anaemia symptoms.	Institute of Hematology & Blood Diseases Hospital, China	Not Applicable	Recruiting
		NCT04390971	ET–01: *Ex vivo* CRISPR-Cas9 therapy targeting the BCL11A enhancer in autologous CD34+ HSPCs to increase HbF and reduce transfusion needs.	Institute of Hematology & Blood Diseases Hospital, China	Not Applicable	Recruiting
		NCT05577312	BRL–101: *Ex vivo* CRISPR-Cas9 therapy using autologous CD34+ hHSPCs to evaluate safety and efficacy.	Bioray Laboratories	Phase 1	Active
		NCT03655678	CTX001: *Ex vivo* CRISPR-Cas9 therapy targeting the BCL11A enhancer in CD34+ HSPCs to increase HbF levels. (Casgevy)	Vertex Pharmaceuticals Incorporated	Phase2/3	FDA Approved
		NCT04925206	ET–01: *Ex vivo* CRISPR-Cas9 therapy using autologous CD34+ HSPCs to disrupt the BCL11A enhancer, increasing HbF levels.	EdiGene (GuangZhou) Inc.	Phase 1	Active
		NCT05356195	TX001: *Ex vivo* CRISPR-Cas9 therapy using autologous CD34+ HSPCs to disrupt the BCL11A enhancer, increasing HbF levels.	Vertex Pharmaceuticals Incorporated	Phase 3	Recruiting
		NCT06065189	CS–101: *Ex vivo* base editing therapy using autologous CD34+ haematopoietic stem cells to disrupt BCL11A, increase HbF, and reduce transfusion needs	Institute of Hematology & Blood Diseases Hospital, China	Phase 1	Recruiting
		NCT06328764	CS–101: *In vitro* Transformer Base Editing (tBE) therapy targeting the BCL11A binding site in the HBG promoter to increase HbF production and compensate for deficient adult haemoglobin (HbA).	CorrectSequence Therapeutics Co., Ltd.	Phase 1	Active
		NCT06024876	CS–101: *In vitro* tBE therapy targeting the BCL11A binding site in the HBG promoter to increase HbF levels.	CorrectSequence Therapeutics Co., Ltd.	Phase 1	Recruiting
		NCT06291961	CS–101: *In vitro* base editing therapy targeting the BCL11A binding site in the HBG promoter to reactivate γ-globin production, increase HbF levels, and reduce anaemia symptoms.	CorrectSequence Therapeutics Co., Ltd.	Phase 1	Recruiting
	Hereditary Angioedema (HAE)	NCT06634420	NTLA–2002: CRISPR-Cas9 to knock out the KLKB1 gene in the liver, reducing plasma kallikrein production to lower the frequency and severity of HAE attacks.	Intellia Therapeutics	Phase 3	Recruiting
		NCT05120830	NTLA–2002: *In vivo* CRISPR-Cas9 therapy delivered via lipid nanoparticles to knock out the KLKB1 gene, reducing bradykinin production.	Intellia Therapeutics	Phase 1/2	Active
Infectious diseases	COVID–19 Respiratory Infection	NCT04990557	PD–1 and ACE2 Knockout T Cells: *Ex vivo* CRISPR-Cas9 therapy targeting PDCD1 (PD–1) and ACE2 genes in CD8+ T cells to reverse T-cell exhaustion and enhance long-term immunity.	Mahmoud Ramadan Mohamed Elkazzaz, Kafrelsheikh University	Phase 1/2	Unknown
	Herpes Simplex Virus Infection	NCT04560790	BD111: *In vivo* CRISPR-Cas9 mRNA therapy administered via corneal injection to target HSV–1, aiming to clear viral infection and prevent corneal blindness.	Shanghai BDgene Co.	Not Applicable	Completed
	HIV/AIDS	NCT03164135	CRISPR-Cas9 CCR5 Knockout: *Ex vivo* CRISPR-Cas9 therapy targeting CCR5 gene in CD34+ haematopoietic stem cells to confer resistance to HIV–1 infection.	Affiliated Hospital to Academy of Military Medical Sciences	Not Applicable	Unknown
		NCT05144386	EBT–101: *In vivo* CRISPR-Cas9 therapy delivered via intravenous (IV) infusion to target and excise HIV–1 proviral DNA from infected cells.	Excision BioTherapeutics	Phase 1/2	Active
		NCT02388594	ZFN-mediated CCR5 gene knockout in CD4+ T cells to reduce HIV susceptibility, with or without prior cyclophosphamide conditioning.	University of Pennsylvania	Phase 1	Completed
	HPV-Related Malignant Neoplasm	NCT03057912	TALEN and CRISPR/Cas9 targeting HPV16/18 E6/E7 oncogenes to disrupt DNA, induce apoptosis, and inhibit cell growth.	Sun Yat Sen University Hospital	Phase 1	Unknown
	HPV16-Positive Cervical Intraepithelial Neoplasia (CIN)	NCT03226470	TALEN (T512) targeting HPV16 E6 and E7 oncogenes to disrupt DNA, decrease E6/E7 expression, and induce apoptosis.	Huazhong University of Science and Technology	Phase 1	Unknown
Neurological disorders	Duchenne Muscular Dystrophy	NCT05514249	CRD-TMH–001: *In vivo* CRISPR-Cas9 therapy via IV infusion to repair a rare DMD gene mutation and restore dystrophin	Cure Rare Disease	Phase 1	Unknown
		NCT06594094	HG302: *In vivo* CRISPR-hfCas12Max therapy delivered via AAV vector to edit the exon 51 splice donor site, restoring dystrophin expression.	HuidaGene Therapeutics Co., Ltd.	Not Applicable	Recruiting
		NCT06392724	GEN6050X: *In vivo* base editing therapy delivered via dual AAV9 vectors to skip exon 50, restoring dystrophin expression.	Peking Union Medical College Hospital	Phase 1	Recruiting
	MECP2 Duplication Syndrome	NCT06615206	HG204: *In vivo* CRISPR-hfCas13Y RNA-editing therapy delivered via intracerebroventricular injection to knock down MECP2 mRNA, reducing protein levels and improving symptoms.	HuidaGene Therapeutics Co., Ltd.	Not Applicable	Recruiting
Oncology	Mesothelin Positive Tumours	NCT03747965	CRISPR-Cas9 edited CAR-T cells with PD–1 gene knockout, targeting mesothelin, combined with paclitaxel and cyclophosphamide preconditioning.	Chinese PLA General Hospital	Phase 1	Unknown
		NCT03545815	CRISPR-Cas9 edited CAR-T cells with PD–1 and TCR gene knockout, targeting mesothelin to enhance anti-tumour immunity and persistence.	Chinese PLA General Hospital	Phase 1	Unknown
		NCT03747965	PD–1 Knockout CAR-T Cells: *Ex vivo* CRISPR-Cas9-engineered chimeric antigen receptor (CAR-T) cells targeting mesothelin, combined with paclitaxel and cyclophosphamide to modulate the tumour microenvironment.	Chinese PLA General Hospital	Phase 1	Completed
		NCT03545815	CRISPR-Cas9 edited CAR-T cells: Targeting mesothelin with PD–1 and TCR gene knockout to enhance immune response and tumour clearance.	Chinese PLA General Hospital	Phase 1	Unknown
	Relapsed/Refractory Multiple Myeloma	NCT05722418	CB–011: Allogeneic CRISPR-Cas9-engineered CAR-T cells targeting B cell maturation antigen (BCMA) to enhance anti-tumour activity.	Caribou Biosciences, Inc.	Phase 1	Recruiting
	Esophageal Cancer	NCT03081715	PD–1 Knockout T Cells: *Ex vivo* CRISPR-Cas9 engineered autologous T cells with PD–1 gene knockout, infused to enhance immune response against cancer.	Hangzhou Cancer Hospital	Not Applicable	Completed
	Breast Cancer	NCT05812326	AJMUC1: *Ex vivo* CRISPR-Cas9-engineered CAR-T cells with PD–1 gene knockout, targeting MUC1, to improve immune response and tumour clearance	Sun Yat-Sen Memorial Hospital	Phase 1/2	Completed
		NCT05812326	AJMUC1 PD–1 gene knockout anti-MUC1 CAR-T cells targeting aberrantly glycosylated MUC1. Dose escalation to identify MTD/MAD.	Sun Yat-Sen Memorial Hospital of Sun Yat-Sen University	Phase 1/2	Completed
	HPV-Related Cervical Intraepithelial Neoplasia I	NCT03057912	TALEN and CRISPR-Cas9: Genome editing therapies targeting HPV16 and HPV18 E6/E7 DNA to decrease oncogene expression, induce apoptosis, and inhibit lesion growth.	Sun Yat-Sen Memorial Hospital	Phase 1	Unknown
	Relapsed/Refractory Haematologic Malignancies	NCT06492304	CTX131: *Ex vivo* CRISPR-Cas9-engineered allogeneic CAR-T cells targeting CD70, designed to enhance tumour clearance and immune response.	CRISPR Therapeutics AG	Phase 1/2	Recruiting
	Relapsed/Refractory B-cell Malignancies	NCT05643742	CTX112: *Ex vivo* CRISPR-Cas9-engineered allogeneic CAR-T cells targeting CD19, designed to enhance immune-mediated tumour clearance.	CRISPR Therapeutics AG	Phase 1/2	Recruiting
	Relapsed/Refractory Solid Tumours	NCT05795595	CTX131: *Ex vivo* CRISPR-Cas9-engineered allogeneic CAR-T cells targeting CD70, designed to enhance tumour clearance and immune response.	CRISPR Therapeutics AG	Phase 1/2	Recruiting
	Relapsed/Refractory B-cell Non-Hodgkin Lymphoma	NCT04637763	CB–010: *Ex vivo* CRISPR-edited allogeneic CAR-T cells targeting CD19, combined with cyclophosphamide and fludarabine lymphodepletion.	Caribou Biosciences, Inc.	Phase 1	Recruiting
	Relapsed/Refractory CD19+ Leukaemia or Lymphoma	NCT04037566	XYF19 CAR-T Cells: *Ex vivo* CRISPR-edited autologous CAR-T cells targeting CD19 with HPK1 gene knockout to enhance anti-tumour activity, combined with cyclophosphamide and fludarabine preconditioning.	Xijing Hospital	Phase 1	Recruiting
	Pleural Malignant Tumours	NCT06726564	MT027: Locoregional delivery of CRISPR-Cas9 engineered CAR-T cells targeting B7H3, administered via intrapleural injection to enhance tumour clearance.	Suzhou Maximum Bio-tech Co., Ltd.	Phase 1	Recruiting
	Brain, Meninges, and Spinal Cord Metastatic Tumours	NCT06742593	MT027: Off-the-shelf CRISPR-engineered allogeneic CAR-T cells targeting B7H3, administered via intraventricular or intrathecal injection, to reduce tumour burden and minimise host immune reactions.	Suzhou Maximum Bio-tech Co., Ltd.	Phase 1	Not Yet Recruiting
	Metastatic Gastrointestinal Epithelial Cancer	NCT04426669	CRISPR-Cas9 edited Tumour-Infiltrating Lymphocytes (TIL): Targeting CISH gene knockout to enhance T-cell anti-tumour activity, combined with cyclophosphamide, fludarabine, and aldesleukin.	Intima Bioscience, Inc.	Phase 1/2	Recruiting
	Advanced Hepatocellular Carcinoma	NCT04417764	PD–1 Knockout T Cells: *Ex vivo* CRISPR-Cas9-engineered autologous T cells with PD–1 gene knockout, infused via percutaneous liver puncture. Administered in combination with transarterial chemoembolisation (TACE).	Central South University	Phase 1	Recruiting
	Metastatic Non-Small Cell Lung Cancer	NCT05566223	CISH Inactivated TILs: *Ex vivo* CRISPR-Cas9-engineered Tumour-Infiltrating Lymphocytes (TILs) with CISH gene knockout, combined with fludarabine and cyclophosphamide.	Intima Bioscience, Inc.	Phase 1/2	Not Yet Recruiting
	Recurrent or Progressive High-Grade Glioma	NCT06737146	MT027: Locoregional delivery of CRISPR-engineered allogeneic CAR-T cells targeting B7H3, administered in escalating doses to evaluate safety and efficacy.	Suzhou Maximum Bio-tech Co., Ltd.	Phase 1	Not Yet Recruiting
	Esophageal Cancer	NCT03081715	PD–1 Knockout T Cells: *Ex vivo* CRISPR-Cas9-engineered autologous T cells with PD–1 gene knockout, administered in two cycles to enhance immune response.	Hangzhou Cancer Hospital	N/A	Completed
	B Acute Lymphoblastic Leukaemia	NCT04557436	PBLTT52CAR19: Allogeneic CRISPR-Cas9-engineered CAR-T cells targeting CD19+ leukaemia, engineered to knock out CD52 and TRAC loci for enhanced efficacy.	Great Ormond Street Hospital for Children NHS Foundation Trust	Phase 1	Active
	B Cell Leukaemia, B Cell Lymphoma	NCT03398967	Allogeneic CAR-T cells targeting CD19 and CD20 or CD22, engineered via CRISPR-Cas9 to enhance anti-tumour activity.	Chinese PLA General Hospital	Phase 1	Unknown
		NCT03166878	Allogeneic CD19-directed CAR-T cells (UCART019) engineered via CRISPR-Cas9 to disrupt TCR and B2M genes, reducing GVHD and enhancing persistence.	Chinese PLA General Hospital	Phase 1/2	Unknown
	Metastatic Non-small Cell Lung Cancer	NCT02793856	PD–1 Knockout T Cells: *Ex vivo* CRISPR-Cas9 engineered autologous T cells with PD–1 gene knockout, combined with cyclophosphamide to enhance immune response.	Sichuan University and Chengdu MedGenCell Co., Ltd.	Phase 1	Completed
	EBV-Associated Advanced Malignancies	NCT03044743	PD–1 Knockout EBV-CTLs: Autologous CRISPR-Cas9-engineered T cells targeting PD–1 to enhance anti-tumour immunity in EBV-associated malignancies.	Yang Yang	Phase 1/2	Completed
	Relapsed/Refractory Acute Myeloid Leukaemia	NCT06128044	CB–012: Allogeneic CRISPR-Cas9-engineered CAR-T cells targeting C-type lectin-like molecule–1 (CLL–1) to enhance anti-tumour activity.	Caribou Biosciences, Inc.	Phase 1	Recruiting
		NCT05662904	Donor-derived CD34+ HSC with CRISPR/Cas9-mediated CD33 deletion: Designed to enhance resistance to CD33-directed immunotherapy	German Cancer Research Center	Phase 1	Not Yet Recruiting
		NCT03190278	UCART123v1.2: T-cell therapy targeting CD123 in AML. Administered via intravenous infusion, dose escalation is used.	Cellectis S.A.	Phase 1	Recruiting
	Relapsed/Refractory B-cell Non-Hodgkin Lymphoma (B-NHL)	NCT05607420	TALEN-engineered UCART20x22 cells administered intravenously; dose-finding and dose-expansion to determine MTD and RP2D.	Cellectis S.A.	Phase 1/2	Recruiting
	Stargardt Disease, Cone-Rod Dystrophy, Juvenile Macular Degeneration	NCT06467344	ACDN–01: Base-editing therapy administered as a single subretinal injection. Open-label, single ascending dose study to evaluate safety and preliminary efficacy.	Ascidian Therapeutics, Inc.	Phase 1/2	Recruiting
	Relapsed/Refractory B-cell ALL	NCT04150497	UCART22: Allogeneic T-cell therapy targeting CD22, intravenous infusion, dose escalation.	Cellectis S.A.	Phase 1/2	Recruiting
	Cervical Intraepithelial Neoplasia (CIN)	NCT02800369	ZFN–603 and ZFN–758: ZFN targeting HPV16/18 E7, inducing DNA cleavage and apoptosis in HPV-positive cells.	Huazhong University of Science and Technology	Phase 1	Active
	T-Cell Acute Lymphoblastic Leukaemia (T-ALL), T-Cell Lymphoblastic Lymphoma (T-LL)	NCT05885464	BEAM–201: Multiplex base-edited, allogeneic anti-CD7 CAR-T cells, targeting CD7 in T-ALL and T-LL.	Beam Therapeutics Inc.	Phase 1/2	Active

### New technology in progress

#### Prime editing and CRISPR-Cas effectors as next-generation CRISPR technologies

Emerging technologies in genome editing are pushing the boundaries of genetic engineering, eliminating or reducing several limits associated with the therapeutic applicability of the conventional CRISPR technology, offering more precise, versatile and efficient tools for manipulating genetic material. Among these innovations are prime editing (PE) and approaches involving CRISPR-Cas effectors such as Cas12 and Cas13, each representing a significant step forward in their respective fields.

**Figure 9.**
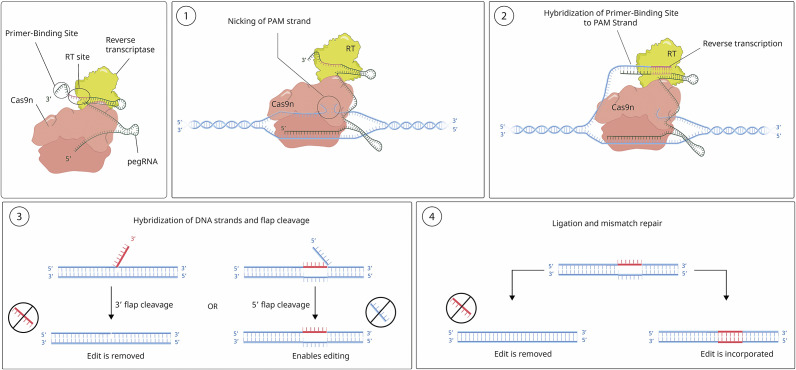
Mechanism of Prime Editing Approach (Ref 140). Cell transfection involves introducing both the pegRNA and the fusion protein for genomic editing. This is typically achieved by delivering vectors into the cells. Once inside, the fusion protein initiates genomic editing by cleaving the target DNA sequence, revealing a 3′-hydroxyl group. This group serves as the starting point (primer) for the reverse transcription of the RT template section of the pegRNA. This process gives rise to an intermediate structure that branches out, featuring two DNA flaps: a 3′ flap containing the freshly synthesised (edited) sequence and a 5′ flap holding the unnecessary, unedited DNA sequence. Subsequently, structure-specific endonucleases or 5′ exonucleases cleave the 5′ flap. This sequential process facilitates the ligation of the 3′ flap, resulting in a heteroduplex DNA comprised of one edited strand and one unedited strand. The reannealed double-stranded DNA exhibits nucleotide mismatches at the editing site. To rectify these mismatches, cells utilise the inherent mismatch repair mechanism, which leads to two potential outcomes: (i) the information in the edited strand is replicated into the complementary strand, thus permanently incorporating the edit; (ii) the original nucleotides are reintegrated into the edited strand, effectively excluding the edit.

PE is a breakthrough technology that goes beyond traditional CRISPR-Cas9 by offering unprecedented precision in genome editing (Ref 140). It is the first precise genome-editing approach, allowing all 12 possible base-to-base conversions, plus insertions or deletions, with minimised off-target effects (Figure 9). It directly rewrites the target DNA sequence without relying on DSBs or donor DNA templates and functions without the need for a precisely positioned PAM sequence for nucleotide targeting, offering more flexible and precise editing (Ref 141). The PE guide RNA (pegRNA) not only guides Cas9 to the target DNA but also provides the necessary template for the insertion, deletion, or conversion of specific DNA sequences. Since the report of the initial version, several new generations and variants of PE have been developed to enhance efficiency through modification of the involved Cas9 and RT enzymes, the pegRNA/sgRNA combination, the structure of the pegRNA, and host protein expression regulation via epigenetic mechanisms (Ref 142). Despite its precision, at its early stage of development, PE still faces challenges in terms of efficiency and delivery, and a universal PE mechanism needs to be optimised. G1 state was shown to be the most suitable step for cell modification for the PE process. Adjusting the endogenous host factors to make the cells permissive for this editing is listed among the future challenges. Other challenges also remain, such as establishing an optimised universal vector for the delivery of the large PE complexes along with the long pegRNAs and regulatory elements and managing immunity, particularly against the pathogen-associated molecular patterns (PAMPs) possessed by the components of the PE machinery (Ref 142). Overall, PE is still a newer technology, relatively in its infancy, that may require additional optimisation and expertise to be transferred fully and effectively into the clinic (Ref 140). The first clinical trial application involving a prime editor received FDA clearance in April 2024 (Refs 143, 144). The study is held by Prime Medicine, Inc., and is structured as an open-label, single-arm, multicenter Phase 1/2 study testing the efficacy and safety of the transplantation of *ex vivo*-modified prime-edited autologous CD34+ stem cells (PM359) in autosomal recessive Chronic Granulomatous Disease (CGD) caused by *NCF1* (Neutrophil Cytosolic Factor 1) gene mutations (NCT06559176). The company is developing PE-based strategies for several other diseases, including X-linked CGD, Wilson’s disease, and cystic fibrosis, as specified in their pipeline.

In the realm of genome engineering, the term ‘CRISPR’ or ‘CRISPR-Cas’ is commonly employed as a broad reference encompassing various systems such as CRISPR-Cas9, Cas12, Cas13 and others. These systems are programmable to target specific genetic code stretches, enabling precise DNA editing and serving diverse purposes, including the development of new diagnostic tools with over 200 engineered variants currently present. Cas12 effectors (also known as Cpf1) exhibiting a variety of sizes, PAM requirements, substrate recognition patterns and interference mechanisms were classified as a unique type V CRISPR–Cas system following the discovery of the Cas12a nuclease as an alternative to Cas9 (Ref 145). More than a dozen distinct Cas12 subtypes have reported since (Ref 146). They share many features with Cas9 but have some key distinctions, such as the DNA-cutting mechanism. Unlike Cas9, which makes a blunt cut across both strands of DNA, Cas12 generates staggered (sticky) ends, which can facilitate more precise integration of foreign DNA into the genome. This characteristic is particularly useful for certain types of genome-editing applications, such as gene knock-ins, where inserting a gene is more efficient with staggered cuts. Also, Cas12 recognises a different PAM than Cas9; while Cas9 typically requires a 5′-NGG-3′ PAM sequence, Cas12 recognises a 5′-TTTV-3′ PAM, where V is any base except for T. This expands the range of targetable genomic sequences, offering additional flexibility where Cas9 may not work as effectively. Cas12 exhibits higher specificity for its target DNA compared to Cas9, which can reduce off-target effects. Additionally, Cas12 has a collateral cleavage activity; once it cuts its target DNA, it can cleave single-stranded DNA non-specifically, which could have potential applications in diagnostics and biosensing. The Doudna lab employed Cas12a’s non-specific single-stranded DNA degradation to establish the DNA Endonuclease Targeted CRISPR Trans Reporter method, referred to as DETECTR (Ref 147). It leverages the indiscriminate cleavage and degradation of nearby ssRNA and single-stranded DNA (ssDNA), which triggers the cleavage and activation of a reporter. The observable signal produced by this reporter can be assessed and measured, allowing for the identification and quantification of the presence of DNA, RNA, or a specific mutation. In summary, CRISPR-Cas12 expands the range of editable genomic sites and offers distinct advantages for precise DNA insertion and lower off-target effects, making it a promising tool for genome engineering, gene therapy and synthetic biology, already utilised in various clinical trials (Table 1). While constituting a breakthrough in human gene editing, the immunogenicity of the Cas effectors remains a problem to be solved via advanced protein engineering and/or improved delivery systems (Refs 78, 145).

CRISPR-Cas13 is a single-strand RNA-targeting genome-editing tool, distinguishing itself from other CRISPR systems like Cas9 and Cas12, which target DNA (Ref 148). It can be programmed to work on specific RNA molecules for degradation or modification without modifying the genomic DNA, for induction of temporary changes to RNA or when DNA editing may be challenging. By enabling RNA-specific editing, CRISPR-Cas13 adds a new dimension to genetic engineering, allowing post-transcriptional alteration of gene expression to explore gene regulation mechanisms, developing RNA-based therapies and improving diagnostics. Through its *in vitro* collateral activity, Cas13 not only specifically cleaves its target RNA but also indiscriminately degrades any nearby RNA, which makes it useful for diagnostic applications to develop quick and highly sensitive nucleic acid detection methods (Refs 149, 150). This has been used in various platforms for rapid and sensitive detection of RNA pathogens, such as viruses. The Zhang lab recently introduced the Specific High Sensitivity Enzymatic Reporter UnLOCKING methods, known as SHERLOCK and SHERLOCKv2, for *in vitro* precise diagnostics which aim to provide quick, multiplexed ultra-sensitive detection of RNA or DNA in relevant samples (Refs 149, 151). SHERLOCK uses the Type VI CRISPR system (Cas13a), while SHERLOCKv2 utilises types III, V and VI (Csm6, Cas12a and Cas 13, respectively) for improved efficiency in a single reaction to detect four different DNA or RNA fragments (Ref 152). Furthermore, Cas13-mediated approaches are suitable for use in various treatment approaches. A CRISPR/Cas13-mediated RNA targeting therapy (HG202) against neovascular age-related macular degeneration (nAMD) is currently recruiting patients for an early phase 1 study (SIGHT-1; NCT06031727). Perturbation of vascular endothelial growth factor (VEGF) is given as the primary cause of nAMD, where overexpression of VEGF results in the abnormal growth of choroidal neovascularisation (CNV). HG202 employs a single AAV vector to partially reduce VEGFA expression to inhibit CNV formation in AMD patients. Besides the great potential, unforeseen risks and effects remain in relation to the collateral activity of Cas13. Other challenges include the requirement for optimisation of delivery systems and potential immunotoxicity and off-target effects *in vivo* with long-term, constitutive expression of Cas13 proteins (Ref 153).

#### Advancements in high-fidelity and PAM-expanded Cas9 variants for precision genome editing

It is crucial in CRISPR approaches to minimise off-target effects while maintaining/elevating gene-editing accuracy. To address this issue, High-Fidelity and Enhanced Specificity Variants were produced by altering the protein’s interactions with the target DNA, thus increasing specificity without compromising efficiency. Recently, SpCas9-HF1 (High-Fidelity 1) was designed to address off-target effects observed with the wild-type SpCas9, which occasionally binds and cleaves DNA sequences with partial mismatches (Ref 154). Four key mutations (N497A, R661A, Q695A and Q926A) were introduced into the REC1 and RuvC nuclease domains to weaken hydrogen bonds with the DNA backbone, reducing non-specific interactions between Cas9 and the target DNA, and enhancing the requirement for perfect base-pairing between the guide RNA (gRNA) and target DNA by increasing the stringency of DNA binding. SpCas9-HF1 retains high on-target cleavage efficiency similar to wild-type SpCas9 and significantly reduces off-target activity across various genomic loci, suitable for precision genome editing as well as for high-specificity studies in functional genomics to minimise unintended gene perturbations.

Another high-fidelity Cas9 variant is the Enhanced Specificity Cas9 (eSpCas9), which bears three mutations (K848A, K1003A and R1060A) that destabilises the R-loop formation to weaken the interaction between Cas9 and the non-target DNA strand, thus increasing the dependency on precise base pairing between gRNA and the target DNA (Ref 155). eSpCas9 offers increased specificity compared to the wild-type SpCas9 without compromising on-target efficiency and reduces off-target effects. Serving for the same purpose, Hyper-Accurate Cas9 (HypaCas9) was engineered to further improve specificity by altering the conformational dynamics of the HNH nuclease domain (Ref 156). Mutations (N692A, M694A, Q695A and H698A) impact the HNH domain responsible for cleaving the target DNA strand, increasing the requirement for perfect gRNA-DNA matching for HNH domain activation. It provides superior specificity compared to both SpCas9-HF1 and eSpCas9, maintains robust on-target activity, and is highly effective in minimising off-target cleavage across complex genomes.

PAM-Expanded Variants, on the other hand, expand the range of targetable genomic sites by recognising alternative PAM sequences, increasing the flexibility of CRISPR-Cas9 systems. SpCas9-NG was developed to recognise a more permissive PAM, overcoming the limitation of wild-type SpCas9’s strict requirement for the 5′-NGG-3′ PAM sequence (Ref 157). Engineered through structure-guided mutagenesis, SpCas9-NG alters residues in the PAM-interacting domain to tolerate base variations at the third PAM position. As a result, it recognises the 5′-NG-3′ PAM (e.g., NGA, NGC, NGT), expanding the targetable genomic sites by fourfold. xCas9 was developed to recognise an even wider range of PAMs while maintaining high specificity and reduced off-target effects (Ref 158). It recognises the 5′-NG, GAA, GAT-3′ PAM, allowing targeting at sites with NG, GAA, or GAT PAMs. Engineered through directed evolution and high-throughput screening, xCas9 features mutations in the PAM-interacting domain that enhance flexibility, enabling it to recognise non-canonical PAMs while retaining high specificity. It offers an expanded target range with improved specificity and reduced off-target activity compared to wild-type SpCas9. xCas9 is suitable for genome editing in PAM-restricted regions and for therapeutic gene editing with enhanced specificity.

Recently reported Cas9 variants engineered through extensive mutagenesis and structural analyses with relaxed PAM requirements include SpG-Cas9 (SpG) and SpRY-Cas9 (SpRY) (Ref 159). These variants are capable of targeting almost any genomic sequence. SpG recognises the 5′-NGN-3′ PAM, allowing broad targeting at sites with any third base, while SpRY is nearly PAM-less, with minimal constraints, enabling targeting at virtually any sequence. These variants are particularly useful for editing in genomic regions with restrictive PAM availability and for complex genetic modifications, including multiplexed genome editing, as well as for functional genomics and therapeutic applications. Future developments are likely to focus on further enhancing specificity and minimising off-target effects, expanding PAM compatibility for unrestricted genome targeting, and improving delivery systems for safe and efficient therapeutic applications.

#### Epigenetic regulation, multiple gene editing, and large-scale gene screening via CRISPR technologies

Various innovations associated with CRISPR technologies, such as epigenetic regulation, multiple gene editing, and large-scale gene screening, hold immense promise for transforming medicine and synthetic biology. CRISPR tools can activate (CRISPRa) or interfere with the function of genes (CRISPRi) via transcriptional modulation without altering the DNA sequence (Ref 160). Catalytically inactive Cas9 (dCas9), when fused with effector domains, enables precise activation or repression of target genes. These approaches can be utilised to investigate and modify epigenetic states, providing valuable insights into gene regulation and cellular reprogramming, thus holding great potential for treating diseases with an epigenetic component, including cancer (Ref 161). Reactivating silenced tumour suppressor genes or suppressing oncogenes will be valuable in designing new therapeutic strategies. Epigenetic regulation via CRISPR approaches also enables the reprogramming of cells into desired types, aiding in regenerative medicine and the development of personalised therapies. While the forced ectopic expression of transcription factors is frequently associated with off-target effects and heterogeneous reprogramming, activation of endogenous pluripotency factors via CRISPRa technology may be effective in the reduction of heterogeneity as well as in providing a highly efficient reprogramming process (Ref 162).

The integration of multiple CRISPR-based technologies will open up new possibilities. Combining epigenetic modulation with multiple gene editing may intricately rewire gene networks, paving the way for more advanced synthetic biology applications and therapeutic treatments. Employing CRISPR for gene editing and regulation in organoids will create more precise models of human diseases, advancing drug discovery and personalised medicine. The potential of artificial intelligence to boost gRNA design for precision to cut off-target effects is also remarkable (Ref 163).

CRISPR-based gene screening has revolutionised functional genomics, and future advancements will improve its scale, resolution, and efficiency. Developing more comprehensive gRNA libraries will enable genome-wide studies to uncover gene functions, pathways, and therapeutic targets. Furthermore, combining CRISPR screening with single-cell sequencing technologies will offer unique insights into gene function at the cellular level, enabling researchers to explore tissue heterogeneity and disease variations. Also, advancements in inducible CRISPR systems will enable researchers to study gene function in a dynamic manner, facilitating time-resolved and tissue-specific gene screening. These technologies have already been used in genetic screens to explore gene functions and identify genes involved in various biological pathways. This approach aids in decoding genetic networks and offers crucial insights into severe diseases, with the goal of discovering new treatments through gene and cell therapies (Ref 164).

### An overview of the challenges associated with CRISPR technologies

While genome editing tools like CRISPR-Cas have revolutionised our ability to target and modify specific genomic sequences, their application is not without challenges, as we also specified in relevant sections throughout the text. Overall, reducing off-target effects, refining delivery systems for better efficiency and accuracy, and enhancing the safety of applications are issues that still need to be addressed, along with ethical considerations. Also, possible variability in editing efficiencies and the complexities of certain genomic regions mean that not all sequences can be easily or reliably manipulated.

#### Off-target effects: still an issue

Of all the current challenges in gene editing, precise targeting and minimising or eliminating off-target effects through advanced techniques are considered the most crucial. Off-target effects refer to unintended genetic modifications that occur when genomic regions other than the actual target are edited, which can have serious implications in terms of safety and ethics. Disruption of vital genes or regulatory regions that may lead to unforeseen diseases or functional impairments may occur. When germline editing may be targeted, the risk of passing harmful mutations to future generations due to off-target functioning of the editing machinery is a major concern. In fact, bioethical concerns regarding germline editing focus on two different topics, depending on successful or failed editing (Ref 165). In the case of successful germline editing applications, using genome editing for nontherapeutic purposes for eugenics or enhancement is a major concern (Ref 166). Critics warn that this could lead to the commercialisation of human life, widen social inequalities, or spark genetic competition. It also brings out the concern regarding the source or entity from which informed consent will be obtained for these modifications. On the other hand, in the case of failed germline editing, including the creation of serious off-target effects, the biggest concern is the risk of transferring the deleterious mutations and undesirable changes to the next generations. One other significant consequence in this scenario is mosaicism, arising when the nuclease is not able to edit all copies of the target gene or the cells begin to divide before the genome editing process is finished. This may cause major unwanted alterations, complicating outcomes (Ref 165).

Overall, off-target effects are addressed by different strategies to minimise undesired byproducts in CRISPR-Cas-mediated genome editing, including the use of biased and unbiased *in silico* off-target detection tools, modification and engineering of gRNA, utilisation of improved Cas variants and engineering (e.g., high-fidelity Cas9), employing delivery methods that restrict Cas9 activity to the target tissue, and utilisation of newer approaches such as BEs and PEs (Refs 1, 167). The use of anti-CRISPR proteins is also claimed to reduce off-target modifications without affecting on-target action (Refs 168, 169). DNA repair challenges in CRISPR/Cas9 editing are addressed via engineering of the repair pathways by modulating endogenous mechanisms with small molecules or gene editing to enhance HDR efficiency or mitigate NHEJ activity. Temporal control can be achieved by designing delivery systems that synchronise Cas9 activity with the cell cycle to maximise HDR usage (Ref 170). The use of alternative editing systems, such as BEs and PEs, avoids reliance on DSBs and, consequently, on NHEJ or HDR. Additionally, combinatorial approaches, including cell-type-specific delivery and HDR enhancers, optimise editing outcomes for therapeutic applications.

#### Advances and Challenges in CRISPR Delivery Systems

Overcoming the challenges associated with delivering CRISPR components to target tissues will be necessary for advancing translational research and clinical applications in gene editing. Several informative reviews focus on *in vivo* delivery systems in preclinical and clinical CRISPR gene editing approaches (Figure 3) (Refs 35–37). Here we briefly go over the selected viral and nonviral methods for the delivery of CRISPR machinery to human cells. Viral vectors, with the advantages of high transduction efficiency and stable gene expression, are widely preferred for this purpose (Ref 171). One of the most commonly used viral vectors in CRISPR-mediated approaches is the non-pathogenic adeno-associated viruses (AAVs), which can infect both dividing and non-dividing cells and provide long-term gene expression through an episomal genome, reducing the risk of insertional mutagenesis. However, using AAV vectors for efficient *in vivo* delivery is a challenging task. First of all, their small cargo capacity (~4.7 kb) limits the ability to deliver large Cas proteins, like SpCas9, along with guide RNAs. Also, although AAVs generally have low immunogenicity, one other limitation of AAVs as gene editing vectors is that host immune response may create neutralising antibodies against the viral capsid even at low titers (1:5–1:7), blocking target cell entry (Ref 172). Besides, depending on the serotype and the analysed cohort, AAV seropositivity among humans is given as between 30–80% (Ref 173). Overall, strategies to overcome various obstacles in delivering CRISPR-Cas-based genome editing treatments using AAV vectors include developing smaller payloads and regulatory elements, advancing new sequencing strategies for vector characterisation, and engineering novel capsids with enhanced potency, tissue selectivity and ability to evade pre-existing antibodies (Ref 174).

Lentiviral vectors (LVs) are also among the first viral systems that were adapted for genome-editing applications with proven efficiency and improved safety, as well as a larger cargo capacity than AAVs (Ref 175). In fact, an all-in-one vector design expressing both Cas9 and sgRNAs was quickly established following reports of the CRISPR/Cas system functioning in human cells (Ref 176). One of the issues associated with the use of LV vectors for the delivery of CRISPR components is the relatively elevated levels of off-target effects due to permanent expression of the CRISPR/Cas9 tools provided by the integrating LVs, besides the oncogenic potential. In fact, integrating LVs are more frequently preferred in *ex vivo* applications, such as editing stem cells or T cells before transplantation. As a safer alternative with a very weak integration capability and a similar transduction efficiency, use of integrase-deficient lentiviral systems (IDLVs) has been associated with much lower frequency of indel formation and other off-target effects in CRISPR/Cas9-mediated gene editing (Refs 177, 178).

Adenoviruses are very well-studied viruses, both biologically and clinically, which can carry large payloads and provide high transduction efficiencies as vectors (Ref 179). Adenoviral vectors (AdVs) have been successfully used as non-integrating delivery systems in gene editing strategies, bearing a reduced risk of off-target effects and insertional mutagenesis and offering a reliable delivery mechanism for large transgenes such as designer nucleases in a transient pattern (Refs 180–182). All customised CRISPR machinery could be delivered by a single high-capacity gutless AdV (HC-AdVs) (Ref 183). However, adaptive immune responses against the vector and Cas9 remain an issue, as it lowers the effective viral titer and necessitates higher vector doses, which further amplifies the immune response (Refs 182, 184). Furthermore, the percentage of pre-existing antibodies in human populations is given as around 90% (Ref 185). Possible solutions include employing non-human AdV vectors, using viruses with low seroprevalence, vector engineering through copolymer encapsulation, and altering the vector genome for lowering of immunogenicity and unwanted surface interactions (Ref 186).

Thus, while viral vectors are a preferred means of delivery for CRISPR components, each vector is associated with certain challenges that need to be overcome for optimal efficiency and safety. Producing viral vectors at clinical grade and scale is also costly and complex, with batch-to-batch variability impacting consistency and safety. Engineering tissue-specific promoters or modifying capsids can enhance targeting, but achieving precise *in vivo* delivery remains challenging. Nonviral delivery systems may overcome many limitations associated with viral vectors. Chemical and physical systems are explored. Of the chemical approaches, nanoparticles are frequently preferred, as nano-scale materials (1–100 nm) with distinctive biological features due to their size and surface properties. They are favoured for their modifiable surface and high targeting ability, as well as their biological safety and high packaging capacity (Ref 187). They are suitable to be utilised as vectors for CRISPR systems, as the large size and negative charge of the Cas9 RNP complex hinder its efficient transport across the negatively charged mammalian cell membranes (Ref 188). Cationic LNPs condense the anionic cargo through electrostatic interactions, forming LNPs that can promote endocytosis across the cell membrane (Ref 189). LNPs are utilized in a wide variety of studies as synthetic carriers that encapsulate nucleic acids (e.g., mRNA encoding Cas proteins or guide RNAs), which are able to carry large payloads (Ref 186). Composed of ionizable lipids, cholesterol, phospholipids and polyethylene glycol (PEG)-lipids that self-assemble into stable nanoparticles, LNPs avoid risks associated with viral vectors such as insertional mutagenesis and viral immunogenicity. Delivery efficiency can be increased via chemical modifications to enhance stability, targeting specificity and endosomal escape. However, LNPs are often taken up non-specifically by the liver and spleen due to their interaction with serum proteins, limiting their use for tissue-specific editing. Ongoing research aims to improve the properties of LNPs in terms of cell penetration, precise tissue targeting, endosome escape, toxicity reduction, prevention of degradation, and improved long-term storage stability (Ref 187).

Other chemical approaches for the efficient delivery of CRISPR components include hybrid nanoparticles that enhance stability, cargo capacity, loading efficiency and tissue-specific targeting by combining lipid and polymeric materials (Refs 190, 191). These agents can be customised to respond to stimuli such as pH or temperature, improving controlled release. Extracellular vesicles (e.g., exosomes) offer biocompatible and efficient delivery. They can be engineered to carry CRISPR components and target specific tissues by modifying surface proteins. These emerging strategies offer improved specificity, efficiency and biocompatibility, but challenges related to scalability, cost and regulatory approval remain.

Physical methods such as electroporation, microinjection, and hydrodynamic injection employ physical forces to aid in the intracellular delivery of CRISPR/Cas9 machinery through disruption of the host cellular and nuclear membranes. Electroporation, which is widely used in *in vitro* and *ex vivo* approaches, uses electrical pulses to create temporary pores in the cell membrane, allowing CRISPR components to enter the cell (Ref 192). However, although highly efficient in controlled laboratory settings, achieving targeted *in vivo* delivery via electroporation is challenging; the requirement for specialised equipment, its invasive nature with cell-damaging effects, and the technical limitation of scalability and administration skills limit its clinical use. This approach is typically preferred in *ex vivo* applications, such as editing HSCs or T cells, which are then reintroduced into patients with reduced off-target effects due to transient expression (Refs 187, 193, 194). Thus, despite advancements, several challenges hinder the efficient clinical translation of CRISPR delivery systems, with standardisation of the production processes, ensuring batch consistency, and meeting regulatory requirements still posing significant hurdles to outcome (Ref 195).

#### Other challenges

One important issue in clinical trials involving gene editing approaches is the requirement for careful identification of the causes of problematic outcomes. A sample case is a 27-year-old Duchenne Muscular Dystrophy (DMD) patient who was treated with recombinant adeno-associated virus (rAAV) serotype 9 d*Sa*Cas9 (‘dead’ *Staphylococcus aureus* Cas 9, with inactivated nuclease activity) fused to VP64, which, as a custom CRISPR-transactivator treatment, was designed to upregulate cortical dystrophin (Ref 196). Mild cardiac dysfunction and pericardial effusion, followed by acute respiratory distress syndrome (ARDS), were evident, leading to cardiac arrest 6 days following the application and the death of the patient 2 days later. Researchers correlate the death to an innate immune reaction leading to ARDS following the application of a high-dose rAAV gene therapy for advanced DMD, rather than a response against the CRISPR/Cas9 system itself or the transgene (Ref 197). The preexisting disease underlying the treatment is given as the most likely reason for the fatal AAV toxicity (Ref 198). In the case of AAV, as may be valid for other certain delivery systems as well, while studies in mice showed promising results, findings from human studies thus indicate that high-dose systemic AAV administration and related complications constitute an additional challenge to AAV-CRISPR approaches that need deeper consideration and thorough analysis (Ref 199).

Edited cells’ long-term stability and behaviour, including the risk of malignant transformation, require thorough investigation to guarantee sustained therapeutic benefits. Additionally, scalability poses a significant challenge for CRISPR-based treatments, as producing enough of the treatment to meet the needs of a large population is complex (Ref 200). This challenge arises due to technical hurdles in creating personalised therapies and implementing the treatment regimen and associated costs. Vertex announced a wholesale acquisition cost of 2.2 million USD for Casgevy in the United States. Most individuals affected by SCD or BT cannot access this treatment due to its prohibitive cost and restricted availability (Ref 201). Consequently, high costs will likely limit the accessibility of gene-editing drugs to only a handful of medical centres globally.

With the advancements and extensive ongoing research in gene editing, an even greater understanding and management of human diseases will soon be possible. From inherited disorders like SCD and cystic fibrosis to complex conditions such as cancer, researchers harness the power of CRISPR technologies to explore personalised therapeutic interventions. Treatments will be tailored to individual genetic profiles for enhanced efficacy and reduced side effects. New applications in the field also bring out ethical and regulatory considerations, such as equitable access to innovative therapies and the potential for germline alterations. Continued discussions on the ethical implications of gene editing will be essential for formulating guidelines and regulations to ensure responsible and safe applications. Maintaining public trust through transparent communication about CRISPR’s risks and benefits is crucial, as there is a risk of these technologies to be misued to damage the environment and the society. Legal insights and regulations of genome editing differ in various countries (Ref 202). As these technologies move closer to widespread clinical adoption, regulatory agencies such as the FDA (United States), EMA (Europe), NMPA (China) and others will play a crucial role in setting guidelines for safety, efficacy and ethical compliance. Standardising global regulations and ensuring a balanced approach between innovation and human benefit, patient safety, and ethical responsibility will be essential for the successful integration of these technologies into wide clinical practice (Ref 203). Engaging a diverse group of participants, such as researchers, ethicists, lawmakers and the general public, is essential to guarantee the responsible application of CRISPR technology (Ref 204).

In time, the field will likely witness diverse applications with increased collaborations, which will continue leading to the translation of groundbreaking discoveries into brilliant clinical and practical solutions. Ideal therapies will demonstrate long-term safety and efficacy, as well as being easy to manufacture and administer, making them accessible to more patients. Addressing the current concerns in the field through comprehensive research, clinical validation, robust regulatory frameworks, and international collaboration is imperative to harness the full potential of CRISPR-mediated gene editing technology in the near future.
